# Critical complex network structures in animal gastrointestinal tract microbiomes

**DOI:** 10.1186/s42523-024-00291-x

**Published:** 2024-05-03

**Authors:** Zhanshan (Sam) Ma, Peng Shi

**Affiliations:** 1 Computational Biology and Medical Ecology Lab, Kunming Institute of Zoology, Chinese Academy of Sciences, Kunming, China; 2 Evolutionary and Functional Genomics Lab, State Key Laboratory of Genetic Resources and Evolution, Kunming Institute of Zoology, Chinese Academy of Sciences, Kunming, China; 3 Center for Excellence in Animal Evolution and Genetics, Chinese Academy of Sciences, Kunming, China; 4 Faculty of Arts and Science, Harvard Forest, Harvard University, Cambridge, MA 02138 USA

**Keywords:** Animal gastrointestinal tract microbiomes (AGM), Core/periphery network (CPN), High-salience skeleton network (HSN), Phylogenetic (evolutionary) timeline (PT), Host diets and animal gut microbiomes, *Bacteroidetes*/*Firmicutes* (BF) ratio, *Bacteroidetes*/*Proteobacteria* (B/P) ratio, *Firmicutes*/*Proteobacteria* (F/P) ratio

## Abstract

**Background:**

Living things from microbes to their hosts (plants, animals and humans) interact with each other, and their relationships may be described with complex network models. The present study focuses on the critical network structures, specifically the core/periphery nodes and backbones (paths of high-salience skeletons) in animal gastrointestinal microbiomes (AGMs) networks. The core/periphery network (CPN) mirrors nearly ubiquitous nestedness in ecological communities, particularly dividing the network as densely interconnected core-species and periphery-species that only sparsely linked to the core. Complementarily, the high-salience skeleton network (HSN) mirrors the pervasive asymmetrical species interactions (strictly microbial species correlations), particularly forming heterogenous pathways in AGM networks with both “backbones” and “rural roads” (regular or weak links). While the cores and backbones can act as critical functional structures, the periphery nodes and weak links may stabilize network functionalities through redundancy.

**Results:**

Here, we build and analyze 36 pairs of CPN/HSN for the AGMs based on 4903 gastrointestinal-microbiome samples containing 473,359 microbial species collected from 318 animal species covering all vertebrate and four major invertebrate classes. The network analyses were performed at host species, order, class, phylum, kingdom scales and diet types with selected and comparative taxon pairs. Besides diet types, the influence of host phylogeny, measured with phylogenetic (evolutionary) timeline or “age”, were integrated into the analyses. For example, it was found that the evolutionary trends of three primary microbial phyla (*Bacteroidetes*/*Firmicutes/Proteobacteria*) and their pairwise abundance-ratios in animals do not mirror the patterns in modern humans phylogenetically, although they are consistent in terms of diet types.

**Conclusions:**

Overall, the critical network structures of AGMs are qualitatively and structurally similar to those of the human gut microbiomes. Nevertheless, it appears that the critical composition (the three phyla of *Bacteroidetes*, *Firmicutes,* and *Proteobacteria*) in human gut microbiomes has broken the evolutionary trend from animals to humans, possibly attributable to the Anthropocene epoch and reflecting the far-reaching influences of agriculture and industrial revolution on the human gut microbiomes. The influences may have led to the deviations between modern humans and our hunter-gather ancestors and animals.

**Supplementary Information:**

The online version contains supplementary material available at 10.1186/s42523-024-00291-x.

## Introduction

Organisms, from microbes through animals and plants to humans, aggregate and form communities, which can be abstracted as complex networks, usually with nodes for organisms and edges (links) for their interactions. In the case of animal gastrointestinal microbiomes (AGMs), network nodes can represent microbes on a host animal species, class, or phylum, and the edges can capture not only the interactions among microbial species, but also the influences of the phylogeny of animal hosts through multi-scale network modeling (from species, through class to phylum and kingdom). Such capabilities are particular important for modeling the AGMs because animal microbiomes and their hosts form so-called holobionts, i.e., animal species and their symbiotic microbes. The holobionts are more like superorganisms with hologenomes (i.e., the total genomes/metagenomes carried by the holobionts), and are subject to natural selection and genetic drifts, and the hologenomes can be passed over next generations with reasonable fidelity [5, 59, 60, 65].

Besides host phylogeny, an equally important factors influencing the evolution or coevolution of holobionts is the animal diet types because microbes frequently determine what animals can eat and digest. In fact, the trophic relationships (who eats/digest who) form the backbones of food webs in ecosystems [11, 55, 56]. Specifically, the symbiotic microbes not only can regulate, modulate, and/or alter the various relationships in the food web networks, primarily the competition, predation and cooperation on ecological time scale (e.g., [13, 34, 66]), but also can shape the coevolution between animal microbes and their hosts within the holobionts [5, 59, 60, 65].

In spite of the obvious multi-facet nature of the AGM-phylogeny—food-web relationships, virtually all existing studies in the field have been focused on the influences of phylogeny and diet types on the AGM biodiversity (e.g., [1, 2, 7, 16, 17, 21, 23, 25, 35, 43, 44, 46–51, 63, 64, 67, 68, 72]), virtually all of which investigated prokaryotes diversity but there are also studies on eukaryome (microeukaryotic organisms associated with animal hosts) diversity [6]. In a previous series of articles [41, 42, 44], we have also investigated the AGM diversity [44] and the underlying mechanisms [41, 42], heterogeneity and their scaling patterns across the spectrums of host animal phylogeny (in terms of phylogenetic or evolutionary timeline) and diet types (herbivores, carnivores, omnivores and the other). However, few of the existing studies have involved comprehensive analyses of the *interactions* either from microbial, host, or holobiont perspectives. In the present article, our focus is on modeling the AGM with complex network approaches with the same datasets we previously analyzed, which covers 4903 AGM samples collected from 318 animal species representing all 6 vertebrate classes and 4 major invertebrate classes. There is no doubt that the biodiversity studies of AGM are important, and in the meantime, it is not the most informative in our opinion because it explicitly ignores the interactions among species and is simply an aggregation measure in the form of entropy of species abundance distribution. For example, measuring biodiversity with Shannon entropy or Simpson index is essentially similar to measuring income distribution in economics with arithmetic average or Gini index, either far from ideal. In fact, Simpson index for measuring biodiversity and Gini index can be derived from each other [37, 38]. A major issue in using diversity or Gini indexes is that they treat individuals as discrete entities and ignore their relationships, instead, focusing on the number of entities, and they are moderately better than using simple statistical averages thanks to their using some kind of non-linear weighting schemes inherited from entropy functions.

There have been extensive applications of network science in life sciences since its start, perhaps because, like social networks, interactions among organisms including their components such as cells and neurons, offer ideal testbed for developing and testing the methods of complex networks, primarily developed by mathematicians and physicists [40, 61]. Consequently, there have been a wide range of network models for choosing to apply to the AGM studies, here we choose two of them: the core/periphery network (CPN) and high-salience skeleton network (HSN) [4, 15, 24]. The CPN distinguish network nodes as densely connected core nodes and loosely connected periphery nodes that are sparsely linked to core. It mirrors the virtually ubiquitous nested structures in ecological communities and highlights the heterogeneities of network nodes from node perspective. The HSN distinguishes network edges (links) as “backbones” (consisting of high-salience skeletons) and “rural roads” (consisting of regular or weak links). It mirrors the nearly universal asymmetricities in species (or other taxa) interactions in ecological communities [27, 28] and highlights the heterogeneities of species interaction strengths from edge perspective. Integrated together, the CPN and HSN offer a powerful approach to detecting critical network structures from both node and link perspectives, covering the perspectives of two only elements of any network models. We further build a series of CPN/HSN models on the scales of host animal species, class, phylum, and diet types, which equivalently incorporate the animal phylogeny and diet types into the AGM network models, and enable us to analyze the effects of phylogeny and diet types on the interactions in AGMs in a network setting. The insights from such network analysis cannot be obtained from diversity or heterogeneity analyses with traditional community ecology approaches. Such insights are also of important practical significance. For example, recent conservation of wildlife advocates for the protection of the whole host-microbes, known as holobiont, rather than the animal per se in traditional conservation biology. This is because microbes obviously can influence host physiology, health, behavior, and psychology [8]. Indeed, animal microbiome should be a critical part of disease ecology of zoonoses [45].

In summary, the objective of this study is to gain insights, from multiple host taxonomic scales (species, order, class, phylum, and kingdom, or diet types from host animal perspective, or community/metacommunity and landscape scales from microbial perspective) on the interactions among animal gastrointestinal microbes by building their complex network models. The interactions include both their critical structures (core, periphery nodes and backbones), as well as their effects measured in the network properties. Although critical network structures are indeed critical for AGM functions, network theory stipulates that weak links or periphery nodes in complex networks are important too because it is the weak leaks that are indispensable for stabilizing the network (e.g., [14]), somewhat similar to the relationship between billionaires and middle classes in a national economy. Therefore, we do not ignore periphery or weak links in the AGM networks either. Finally, we also model the relationship between phylogenetic timeline (PT), also known as evolutionary timeline (ET) but different from familiar phylogenetic distance (PD), and network properties to gain quantitative insights on the effects of phylogeny on AGM structures.

## Material and methods

### Animal gastrointestinal tract microbiome (AGM) datasets and phylogenetic timeline (PT)

A total of 6900 samples of animal gastrointestinal tract microbiome (AGM) distributed over 108 published studies were collected to obtain the AGM datasets in the form of OTU tables. The samples cover 5 animal phyla and 19 classes of the animal kingdom. After quality control, we obtained 4903 samples covering 3 primary animal phyla (*Nematoda*, *Arthropoda* and *Chordates*), 10 classes (*Chromadorea*, *Arachnida*, *Malacostraca*, *Insecta*, *Chondrichthyes*, *Actinopteri*, *Amphibia*, *Sauropsida Aves,* and *Mammalia*), 59 orders, 142 families, 261 genera, and 318 species. They represent all six classes of vertebrates and two most important phyla of invertebrates (*Nematoda* and *Arthropoda*), and therefore are of sufficient representativeness of the animal kingdom. A brief summary of the 4903 AGM samples and their host taxa and diet types is presented in Table 1, and detailed information is referred to Table S7, both in Ma et al. [44]. Among the 10 animal classes, *Mammalia* and *Insecta* took the biggest shares of host species including 123 species (1499 samples) and 76 species (979 samples), respectively. According to the diet types, out of the 4903 AGM samples, 1421, 1229, and 1473 samples are from carnivore, herbivore and omnivore groups, respectively. The remaining 320 samples were classified as the “Other” group, and are excluded from the diet-type related analyses in this article. In consideration of the potential data heterogeneity among the 4903 samples, we download the raw 16S-rRNA reads and recomputed the OTU tables using QIIME-2 software (Version 2018.6.0 [3]). With QIIME2, the OTUs are generated with machine learning algorithms that clusters the reads with 100% similarity as an OTU, and the OTU is then mapped to a taxon against the Greengenes database. A total of 473,359 microbial (bacterial) species were obtained from the previously outlined QIIME2 approach.

The phylogenetic timeline (PT), which is also termed evolutionary timeline (ET), is different from well familiar phylogenetic distance (PD). PT or ET can be considered as a proxy of phylogenetic history or evolutionary age of a taxon, with ancient taxon having larger PT value than modern taxon. In contrast, PD represents the divergence time of a pair of taxa, which is not convenient to use in this study. The PT information for the animal hosts is available at: http://timetree.org [32, 71]. In addition, we used the software package APE [53] and GGTREE [73] to visualize the PT information.

### Core-periphery network (CPN) and high salience skeleton network (HSN)

There have been many applications of complex networks in the studies of animal and human microbiomes [12, 22, 37–40, 61, 70]. Arguably, the biggest challenge has to do with the inferences of species correlation relationships from the sequence reads (species abundances). A slightly less challenging but equally important problem is the selection of proper network models, which should be determined by the research objective. To deal with the first challenge, we resort to Friedman and Alm [19] SparCC (Sparse Correlations for Compositional Data) algorithm as briefly interpreted below. As to the second problem, our choice is determined by the research objective of this study—identifying critical network structures (mirroring the heterogenous or nested structures from node or species perspective and/or asymmetricity in species interactions from link perspective in animal gastrointestinal microbiomes).

It should be noted that, with the state-of-the-art metagenomic technology, we can only infer species correlation relationships (see the introduction below) between microbes, which is different from species interaction relationship in strict theoretical sense. Although we use both species correlation and interaction interchangeably in this article, their difference is a reality, and also a limitation.

#### Estimation of correlation coefficients with SparCC framework

To determine the correlation relationships (estimate the correlation coefficients) between OTUs, we used FastSpar software (https://github.com/scwatts/FastSpar), which is a C++ version of the SparCC (https://bitbucket.org/yonatanf/sparcc), originally developed by Friedman and Alm [19]. Both software packages use the same algorithm. SparCC algorithm significantly alleviate a fundamental issue in inferring the correlation relationships with the so-termed compositional data (relative abundances). With compositional data, most standard statistical methods for estimating correlations are biased because the relative abundances must be sum to 100%. Due to this constraint, relative abundances (fractions) are not independent and tend to be negatively correlated, regardless of the true correlation between the OTUs (which should be determined by absolute abundances, but the true abundances are unknown in virtually all sequencing studies). The algorithm was designed with the following features: (1) rather robust to violations of sparsity assumption, (2) does not depend on any particular distribution of the basis variable (absolute or true abundance variable), (3) explicitly infer the correlation between OTU by leveraging the relationship between basis variation and correlation, (4) employed a Bayesian method for estimating the true relative abundances, (5) multiple rounds of iteration are used to minimize sampling noise, (6) the significance of computed correlation is supported by pseudo-*P* value estimated with bootstrap procedure. These characteristics render it more advantageous over some of most commonly used correlation coefficients for relative abundance (compositional) data. Besides significantly alleviate the fundamental issue from the previously explained “compositional effects,” SparCC algorithm is far less influenced by rare species (with extremely low abundances), sequence depth, and unevenness in sample sizes. The FastSpar is 2–3 orders of magnitude faster than the original SparCC thanks to its C++ implementation and parallel computation. In addition, to minimize the spurious effects of extremely rare OTUs, we only kept the OTUs occurring in more than 2% of the AGM samples for building the networks in this study.

Various non-parametric procedures and/or Bayesian approaches are adopted to minimize the noise effects from estimating the variances and OTU fractions, which allow SparCC not only overcoming the theoretical pitfall in estimating the dependency with relative abundance data, but also obtaining more robust algorithm against sparsity, unevenness in sequencing depth and sample sizes.

#### Core-periphery network (CPN)

Borgatti and Everett [4] first proposed the concept of core-periphery network (CPN), which refers to the meso-scale structure in complex network, with the following defining characteristics: (1) there is a core structure consisting of densely interconnected nodes—many intra-block edges; (2) a periphery structure consisting of loosely connected or disconnected nodes—relatively few intra-block edges; (3) periphery is sparsely linked with core—there may be many or relatively few inter-block edges [15, 20]. Kojaku and Masuda [31] suggested that the CPN is primarily a reflection of heterogenous degree distributions in complex networks, with high degree nodes are most likely classified as core and low degree nodes are most likely classified as periphery. They suggested alternative CPN models beyond a single pair of core-periphery dichotomous meso-structure, but we stick to the original CPN model by Borgatti and Everett [4] for their simplicity, which we believe matches the-state-of-art level data quality of AGM datasets. Intuitively, the CPN allows us to determine the importance of AGM species in terms of their connections to other species. In contrast, the high-salience skeleton network (HSN) can determine the significance of AGM interactions (network links or edges) in terms of their correlation strengthens. Integrated together, CPN and HSN can effectively characterize the heterogeneity or asymmetricity of network structures in complex networks from both node and edge perspectives. In other words, they are able to detect the important (critical) meso-scale (i.e., structure larger than the whole network, but smaller than individual nodes) network structures, which are of potentially significant functions roles, such as maintaining network stability, equivalently the stability of microbiota (microbiome).

Formally, let *G* = (*V*, *E*) be an undirected, unweighted graph with *n* nodes (set of V) and *m* edges (set of E), and let *A* = (*a*_*ij*_) be the adjacency matrix of *G*, where *a*_*ij*_ = *1* if node *i* and node *j* are linked and *0* otherwise. Let *δ* be a vector of length *n* with entries of *1* (if a node belongs to core) or *0* (if node belongs to periphery). Further assuming *P* = (*p*_*ij*_) be the adjacency matrix of the ideal or perfect CPN (no intra-periphery connections) of *n* nodes and *m* edges. The determination of the core-periphery structure can be formulated as an optimization to find vector *δ*, such that the following objective function (*ρ*) reaches its maximum, *i.e.,*

1 $$\delta \Leftarrow max \left\{ {\rho = \mathop \sum \limits_{i,j} A_{ij} P_{ij} } \right\}$$

With vector *δ*, which is the product from maximizing the objective function (Eq. 1), it is then a trivial operation to classify nodes in a network as either core or periphery.

The nestedness (*S*) was defined by Lee et al. [33] and the computational code can be found in Ma and Ellison [38]. The formula for computing (S) can also be found as Eq. (13) of Ma and Ellison [38].

#### High-salience skeleton network (HSN)

Grady et al. [24] introduced the concept of link (edge) salience to measure the importance of a network link in terms of the “consensus” of an ensemble of nodes. The salience concept was aimed to deal with the challenge of an open problem in network science—whether or not a generic heterogenous network can be intrinsically segregated into discrete clusters. The problem is equivalently difficult to reducing the number of links in a network, while preserving the nodes because it is hardly possible to disentangle the link heterogeneity from node heterogeneity—both are mixed in a network. As a side note, obviously, the previous CPN adopted the alternative strategy—reducing the node complexity or classifying nodes based on the consensus of links.

Grady et al. [24] link salience concept considers both node degree and link weight, and the link salience then can be used to classify links into distinct groups. The group of links with high salience is termed a high-salience skeleton, and represents the “highway” or backbone of a network, emphasizing its essential nature to the structural integrity (stability) of the network.

The link salience is defined based on the principle of shortest paths in weighted networks. In the case of microbiome networks, Ma and Ellison [38] suggested to use the inverse of correlation coefficients as weights. Three steps are required to compute the salience (*S*_*ij*_) of a link between node *n*_*i*_ and node *n*_*j.*_ First, the shortest path between each pair of nodes in the network is computed, which is the path with the shortest total effective distance based on effective proximity *d*. Second, the effective proximity (*d*) of the link (*i*, *j*) is *d*_*ij*_ = 1/|*ρ*_*ij*_|, where *ρ*_*ij*_ can be SparCC correlation coefficient between nodes *n*_*i*_ and *n*_*j*_. In this step, the shortest-path tree (SPT) tooted at each node *r*, *T*(*r*) is computed. If there are *N* nodes, *T*(*r*) is a symmetric *N* × *N* matrix, if link (*i*, *j*) is part of at least one of the shortest paths, then cell *T*_*ij*_ = 1, otherwise, *T*_*ij*_ = 0. Finally, the third step, one linearly superimposes all SPTs, and obtain the salience (***S***) of the network,

2 $${\varvec{S}} = \left\langle T \right\rangle = \frac{1}{N}\mathop \sum \limits_{r} T\left( r \right)$$

The ***S*** of the network is still a symmetric *N* × *N*, in which cell *S*_*ij*_ represents the salience of link (*i*, *j*). The value of *S*_*ij*_ ranges between 0 to 1. The higher the value of *S*_*ij*_, the link (*i*, *j*) plays more important role in the shortest paths of more nodes, namely, more nodes designating the link as important [24, 62].

In this study, we propose to measure the heterogeneity of salience values with the Hill numbers [26], which is derived from Renyi [57] entropy. The Hill numbers were first introduced into ecology for measuring biodiversity by Hill [26] but received little attention it deserves until recent years thanks to the efforts by Chao et al. [9, 10] and Ellison [18].

**Fig. 1:**
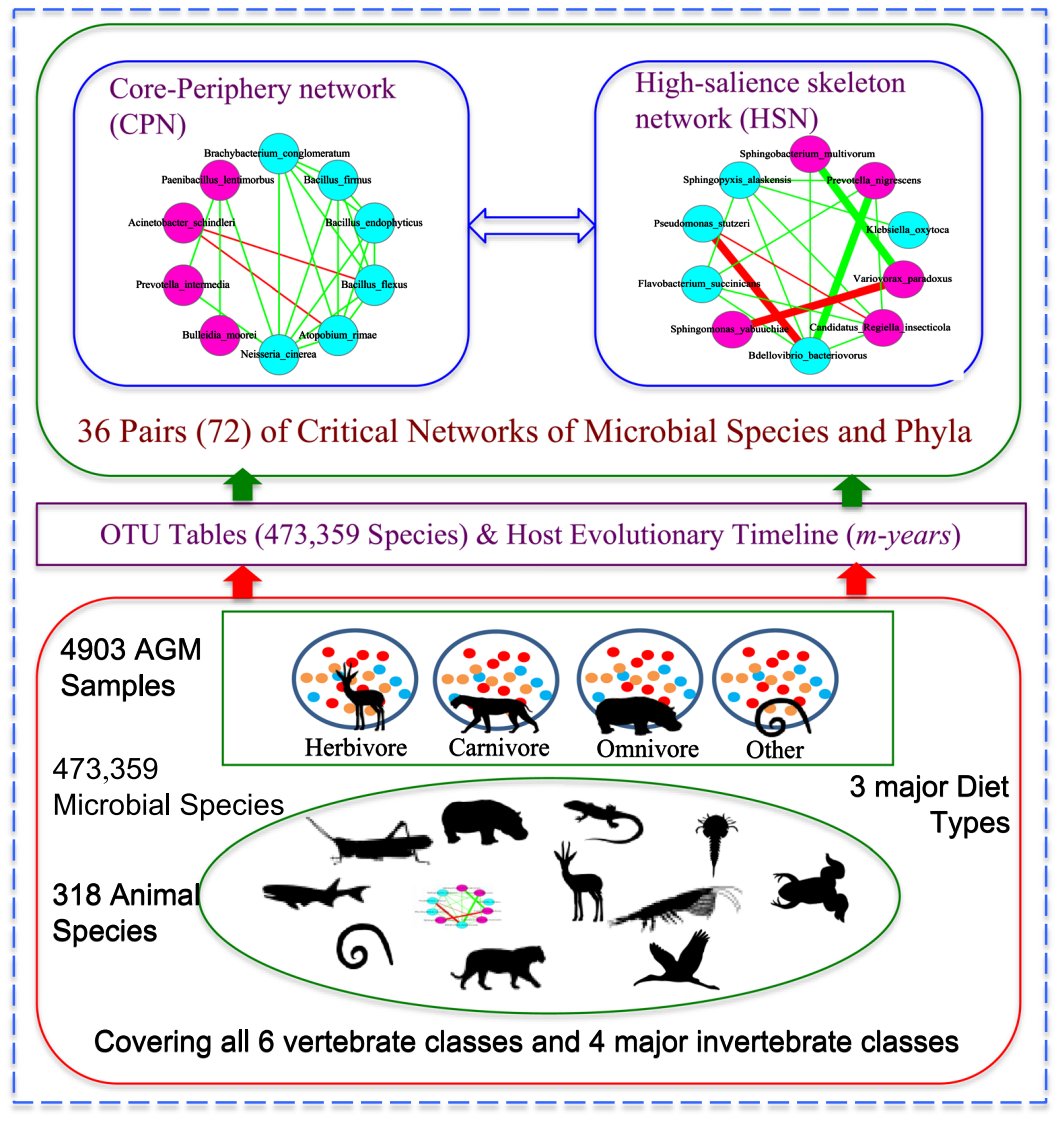
The study design for the AGM (animal gastrointestinal microbiome) network analyses based on the 4903 AGM samples (containing 473,359 microbial species) from 318 animal species. Note that the CPN and HSN in the top section of the figure were drawn with different topology for illustrative purpose. However, in the case of this study, both CPN and HSN for same AGM entity (e.g., host species level) are ‘extracted’ from same species co-occurrence network based on SparCC correlations. Legends: circle in pink represents for core nodes; circle in cyan for periphery nodes; hexagon for network hub; green line for positive correlation; red line for negative correlation; the thick line for high salient skeleton

The CPN/HSN are built upon the OTU correlation network based on the correlation coefficients computed with SparCC algorithm. In other words, the CPN/HSN are critical network structures generated from the OTU correlation networks. The computational codes to fulfill the CPN/HSN detection (analysis) and nestedness metric were developed by Ma and Ellison [37, 38]. The computation code for computing the P/N ratio was developed by Ma and Li [36]. Figure 1 illustrated the study design of this study.

### The overall study design

As illustrated in Fig. 1, we built a total of 49 sets of CPN/HSN networks, with the microbial species as network nodes, corresponding to host animal species, order, class, phylum, kingdom, and diet types. Roja et al. [58] suggested that the host phylogeny and ecology influence the mammalian gut microbiomes differently depend on taxonomic scales. In consideration their finding, we also built another 49 sets of CPN/HSN networks, with the microbial phylum as network nodes, corresponding to the same host taxon and diet type scales as previously. A total of 36 pairs (72) of the CPN/HSN networks were built for the AGM datasets. Due to excessive computational load, at species level, we choose two animal species, *Apis mellifera* and *Bos taurus* as examples to demonstrate the AGM networks at the host species level. Note, that at the class level, four classes with samples less than 200 samples were excluded from the network modeling in consideration of the potential biases from small samples. Somewhat coincidently, the remaining six classes are arguably the most important classes in the animal kingdom. In addition, we perform rigorous statistical tests of major network parameters based on the principle of standard permutation tests [54].

### Relationships between AGM network properties and host evolutionary timelines

We model the relationships between AGM network properties and evolutionary timeline (ET), also known as phylogenetic timeline (PT) by trial-and-error strategy. We adopted two general models: one is the power law (also known as log-linear) model below [Eqs. (3) and (4)], and another is the piece-wise polynomial functions. The power law model is in the form:

3 $$NP = aPT^{b}$$

which is equivalent with the following log-linear model:

4 $$\ln \left( {NP} \right) = \ln \left( a \right) + b \ln \left( {PT} \right)$$

where *NP* is network parameter such as strength of core structures, PT is phylogenetic timeline, *a* and *b* are fitted parameters. The piecewise polynomial functions can be of various forms, for example, the piecewise connection of linear model with another linear model or a quadratic function.

## Results

As illustrated by Fig. 1, we selectively built 14 AGM (animal gastrointestinal microbiome) networks representing various animal taxa (species, class, phylum) and 3 diet types (carnivores, herbivores, and omnivores) given that exhaustively building all possible networks are either computationally too intensive or unnecessary. At animal order level, we exhaustively build all possible AGM networks our datasets can support, i.e., 22 animal-order level AGM networks. These 36 (14 + 22) networks were built at microbial species OTU level. For the 14 non-exhaustive, selected AGM networks, we also build corresponding counterparts at microbial phylum level to facilitate the network analysis since the microbial species-level networks can be too dense to analyze effectively. The network analysis consists of the following main components: (1) core/periphery/skeleton network structures; (2) the strongest network modules (clusters); and (3) the influences of host phylogeny and diet types on the evolution of the critical network structures and on the evolution of key microbial taxa. In the following, we present the findings from the applications of these network analyses according to the network-building schemes outlined previously.

### The core/periphery network (CPN) and high-salience skeleton network (HSN) of the AGMs

Additional file 1: Table S1A presented the basic sample and network information on the 14 selected AGM (animal gastrointestinal microbiome) networks, at microbial species-OTU level, representing various animal taxa and three major diet types (carnivores, herbivores, and omnivores), and Additional file 1: Table S1B presented the microbial phylum OTU-level counterparts of the species-OTU level in Additional file 1: Table S1A. The items included in Additional file 1: Table S1A and S1B include: representative taxa (six animal classes of *Chromadorea, Insecta, Actinopteri, Sauropsida, Aves, Mammalia;* three diet types of carnivores, herbivores, and omnivores; invertebrates vs. vertebrates; two representative animal species of *Apis mellifera* and *Bos taurus*; and all-taxa combined); the numbers of samples used for building each network; numbers of links (edges) and nodes for each network; network density; positive links and negative links and their rations (P/N ratios). The criteria for building these networks, FDR (false discovery rate) control with *P *value = 0.05, and singleton removal, removal of spurious rare OTUs, were also documented in Additional file 1: Table S1. SparCC algorithm was used to compute the OTU correlations, and however, the values of correlation coefficients (*R*) were not used to filter out any network links, other than the associated *P*-values with FDR controls. In other words, all significant correlations were included in the networks, without invoking a cutoff threshold of R. Together with the advantage of SparCC algorithm, the procedures we used to build the AGM networks avoided some issues associated with the traditionally used approaches such as SparCC correlation coefficients.

Both network density and P/N ratios were not found to be significantly correlated with the host phylogenetic timeline (PT) (*P* value > 0.05, Additional file 1: Table S5), suggesting limited influences on host phylogeny on them. The P/N ratios for these AGM networks are exceptionally low compared with results of the human microbiome networks, but caution should be taken since this may simply because the SparCC algorithm recovers significantly more negative correlations as suggested by the algorithm developers [19]. The P/N ratios are mostly less than 1 for higher levels of host taxa (classes and diet types) in Additional file 1: Table S1A, implying more negative correlations in the networks. However, for the animal-order level networks, the PN ratios are mostly larger than 1, implying more positive links. Also for the microbial phylum-OTU level AGM networks of the higher taxa (Additional file 1: Table S1B), the P/N ratios are close to *1*, larger than their microbial species-OTU level counterparts.

Additional file 1: Table S2A and Table S2B formally presented the CPN (core-periphery network) properties including core strength (ρ), ratio of core nodes to the total of core and periphery nodes CP ratio = (C/(C + P)), density matrix of core/periphery structure, P/N ratio in the core/periphery structures, and finally the nestedness (*S*). The most important CPN parameters are arguably the core strength (ρ) and CP ratio. For the microbial species level networks (Additional file 1: Table S2A), the core/periphery structure strength (ρ) suggests that the core/periphery structures are not perfect, and the structure strength is moderate as suggested by the average ρ = 0.228 for the 14 selected higher animal taxa (class and diet types, etc.), and ρ = 0.231 for the 22 animal orders. The average CP ratio of CP = 0.522 for 14 selected AGM networks of the higher level taxa and diet types and of CP = 0.506 for the 22 animal orders indicates that the numbers of core nodes exceed ½ of the total nodes in the networks. For the microbial phylum level networks (Additional file 1: Table S2B), the core/periphery strength (ρ) with average of 0.523 seems to be significantly (twice) higher than that of microbial species level networks, and the CP ratio with average of 0.74 is also higher (approximately ¼ more) that its species level counterparts. Therefore, the core/periphery structures of microbial phylum-level seem to be significantly stronger than microbial species-level counterparts, suggesting the rising core strength with the rise of the taxon aggregation. We postulate that the stronger core/periphery structures with the higher taxon levels may simply be due to the aggregation of taxa, which corresponds to higher level of intra-taxon consistency. The distributions of core/periphery nodes (pink circles for core and cyan circles for periphery nodes) are exhibited in the network graphs to be introduced below.

Additional file 1: Table S3A further exhibited some basic information, for microbial species level AGM networks, on the CPN and HSN (high-salience networks) of 22 animal orders, including the phylogenetic timeline (PT) of each animal order (obtained from http://timetree.org) and number of network links with salience-value exceeding 0.5, which can be considered as high-salience skeletons and are believed to play a critical role in strengthening the critical network structures. Additional file 1: Table S3B further exhibited some basic information, for microbial phylum level AGM networks, on the CPN/HSN of 22 animal orders, including the list of core/periphery phyla in the networks.

Additional file 1: Table S4A and Table S4B presented the HSN properties for the microbial species and phylum level AGM networks, respectively. Those HSN properties include links (%) for percentages of network links with salience > 0; the maximum, mean, median, standard error, skewness, kurtosis of the link salience values; and assortativity. Among these properties, the salience value represents the link strength in the HSN or their importance in the AGM networks. According to the network theory of weak links, while strong links play critical roles in performing the network functions, weak links are not necessarily irrelevant because weak links can stabilize the networks by resisting cascade failures and providing network redundancies. In other words, critical paths consisting of high-salience skeletons, similar to highways in transportation networks, can be more vulnerable to cascade failures without the redundancies offered by weak links.

### The influences of host phylogeny and diet types on the CPN/HSN structures

In the previous section, our focus was on the core/periphery/skeleton (CPS) structures per se, as characterized by various CPS properties. Here, we assess and interpret the influences of host phylogeny and diet types on those CPS properties by analyzing their correlations (SparCC coefficients) with the phylogenetic timeline (PT) (also known as evolutionary timeline or ET).

As shown in Additional file 1: Table S5, all of the CPS network parameters including network density, P/N ratio, core strength (ρ), core density, nestedness (S), link(%) (percentage of links with salience > 0), mean of salience, and median of salience, are not significantly correlated with PT (*P* value > 0.05). That is, we did not find supporting evidence that phylogeny exerts significant effects on the CPS network properties.

**Fig. 2:**
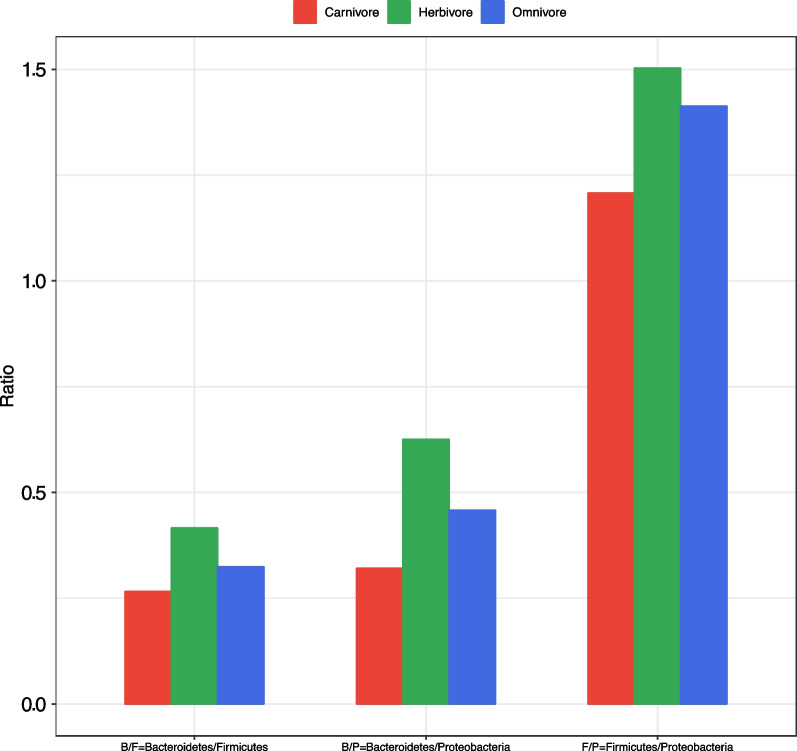
The ratios of B/F (*Bacteroidetes/Firmicutes*), B/P (*Bacteroidetes/Proteobacteria*), and F/M (*Firmicutes/Proteobacteria*)

Next, we look into the relationships between PT and the three important microbial phyla, *Bacteroidetes*, *Firmicutes* and *Proteobacteria* (BFP)*,* as well as their pairwise ratio, BF (*Bacteroidetes*/*Firmicutes*), BP (*Bacteroidetes*/*Proteobacteria*), and FP (*Firmicutes*/*Proteobacteria*) ratios. Additional file 1: Table S6A listed the relative abundances of *Bacteroidetes*, *Firmicutes* and *Proteobacteria*, as well as their pairwise ratios (BF, BP, FP). In terms of the diet types, the BF ratio of herbivores (B/F = 0.416) is indeed the highest, followed by omnivores (B/F = 0.325) and carnivores (B/F = 0.266) (Fig. 2). This trend is obviously consistent with that in the human gut microbiome. The B/P exhibited the same consistent trend with BF and that of the human gut microbiome. The ratio of F/P is also consistent with the previous two ratios (BF/BP). That is, herbivorous animals and vegetarians should have higher B/F, B/P, F/P ratios than carnivores and meatarians, and omnivores sit between them and behind herbivores.

The relationships between different animal orders/classes are more complex since it is often difficult to separate the effects of diet types and phylogeny for most animal taxa. For this reason, we resorted to computing the correlations between PT and the BFP indexes. Additional file 1: Table S6B shows that among the three phyla of BFP (*Bacteroidetes, Firmicutes* and *Proteobacteria*), only the pair of *Firmicutes* and *Proteobacteria* demonstrated consistent negative correlation relationship. The other two pairwise correlations are not statistically significant. This seems to suggest that the rise of *Bacteroidetes* does not necessarily lead to the decline of *Firmicutes* or *Proteobacteria*. Note, that the relationships here in Additional file 1: Table S6B are not necessarily related to the host phylogeny, which is tested in Additional file 1: Table S6C below.

Additional file 1: Table S6C demonstrates that the phylum *Bacteroidetes* or *Firmicutes* appears to be negatively correlated with PT, with Spearman’s correlation coefficient of − 0.289 and − 0.272, respectively, although the correlation may not be statistically significant (*P* value = 0.116 and 0.126 respectively). The relationship between *Proteobacteria* and PT seems to be positive (Spearman = 0.310, *P* value = 0.079), although the correlation is marginally significant (if *P* value = 0.10 is chosen as threshold for judging the significance). Since more recent species has smaller PT value than more ancient species, or we may say that the more recent species is “younger” evolutionarily than the ancient species, the previous relationships between B, F, or P and PT suggests that more recent species appear to have higher *Bacteroidetes* and *Firmicutes* but lower *Proteobacteria* abundances. It should be emphasized that the relationships are not necessarily statistically significant.

As to the evolution of B/F, B/P, and F/P ratios, based on their relationships with the PT values, Additional file 1: Table S6C shows that the B/F-PT relationship is statistically insignificant (*P* value = 0.432). Additional file 1: Table S6C also shows that both B/P and F/P are negatively correlated with the PT with statistical significance of *P* value < 0.01. This seems to suggest that the B/P and F/P ratios of more recent species should be higher, which could be due to either higher *Bacteroidetes* and *Firmicutes* or lower *Proteobacteria* in more recent species, which is a trend explained in the previous paragraph.

Although we cannot make a direct comparison with the results from the human gut microbiome, the evolutionary trends of B/F, B/P, and F/P among animals do not seem to be consistent with the trend in modern human populations. In other words, if we humans continue the natural evolutionary trends of BF/BP, we should have higher BF/BP ratios. The rising of *firmicutes* and *proteobacteria* and the decline of *Bacteroidetes* abundances in the human gut microbiome, especially in obesity populations should be an “artificial selection”, rather than natural selection.

### Further explorations of the AGM with module detection technique

In the final sub-section of the results section, we aim to accomplish two tasks: one is to illustrate the CPN structures with network graphs, and another is to detect strongest modules (most closely connected modules), also known as clusters, in the AGM networks. Since both the tasks use the same network graphs, we delay the illustration of the CPN structures from previous sub-sections to here. We use the MCODE software package to detect the significant modules (clusters) in the AGM networks.

**Fig. 3:**
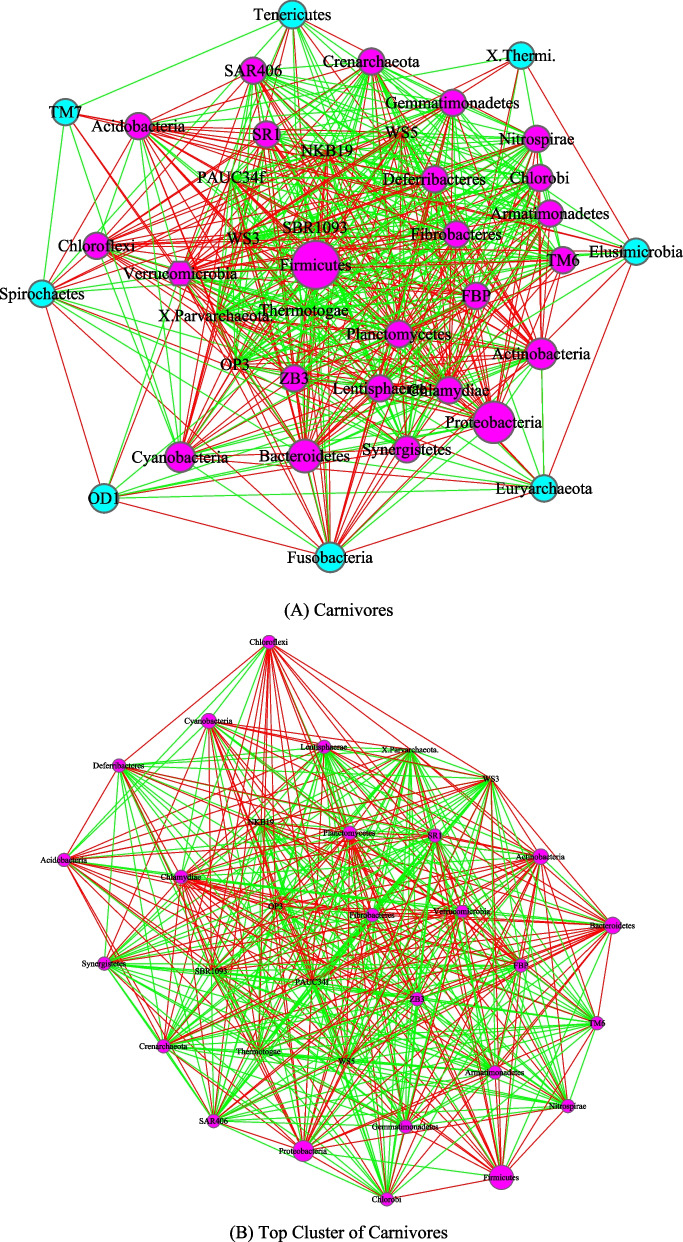

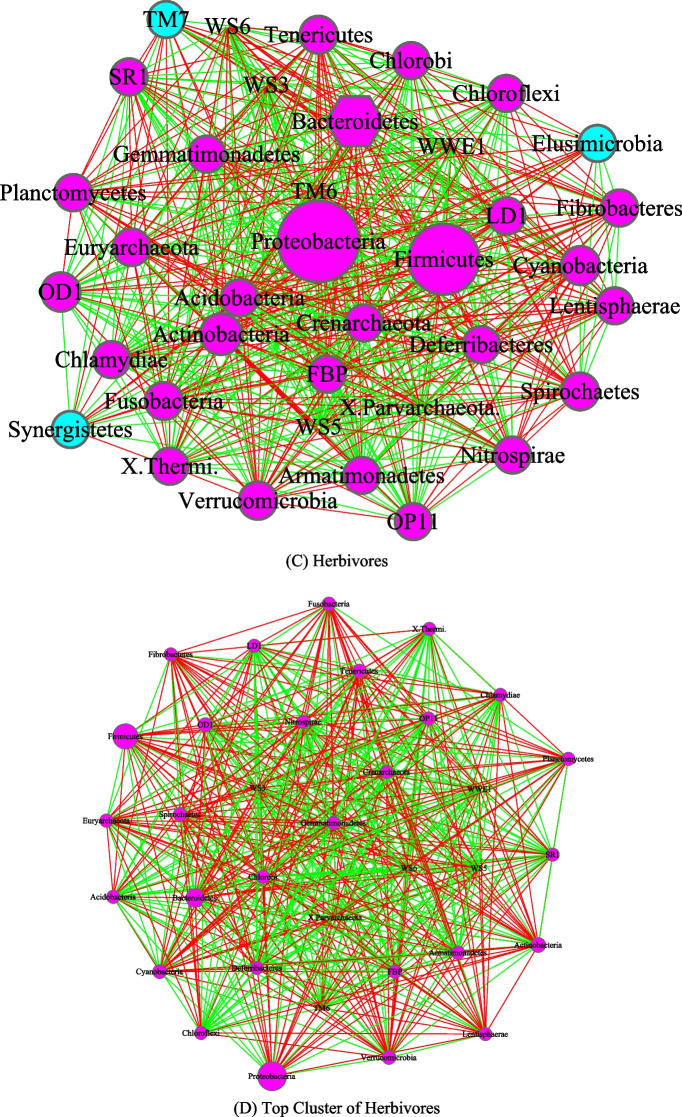

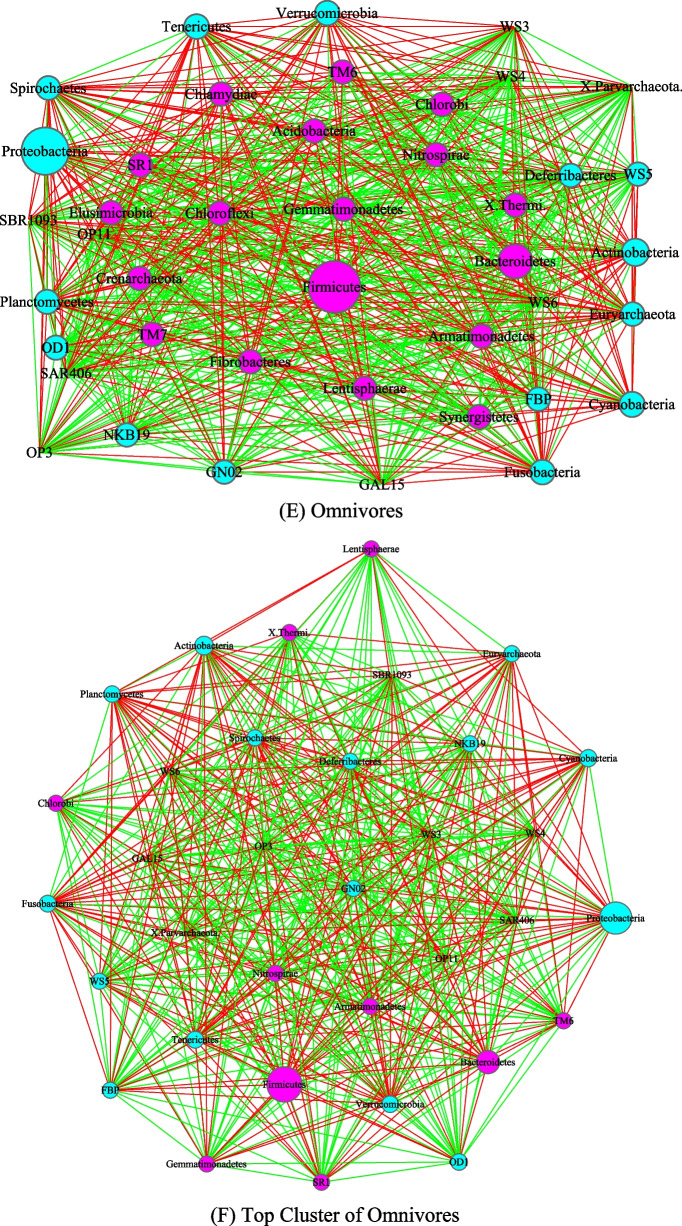
The core/periphery networks (CPN) of the three diet types and their top clusters: **A** carnivores; **B** top cluster of carnivores; **C** herbivores; **D** top cluster of carnivores; **E** omnivores; **F** top cluster of omnivores. Legends: circle in pink represents for core nodes; circle in cyan for periphery nodes; hexagon for network hub; green line for positive correlation; red line for negative correlation

**Fig. 4:**
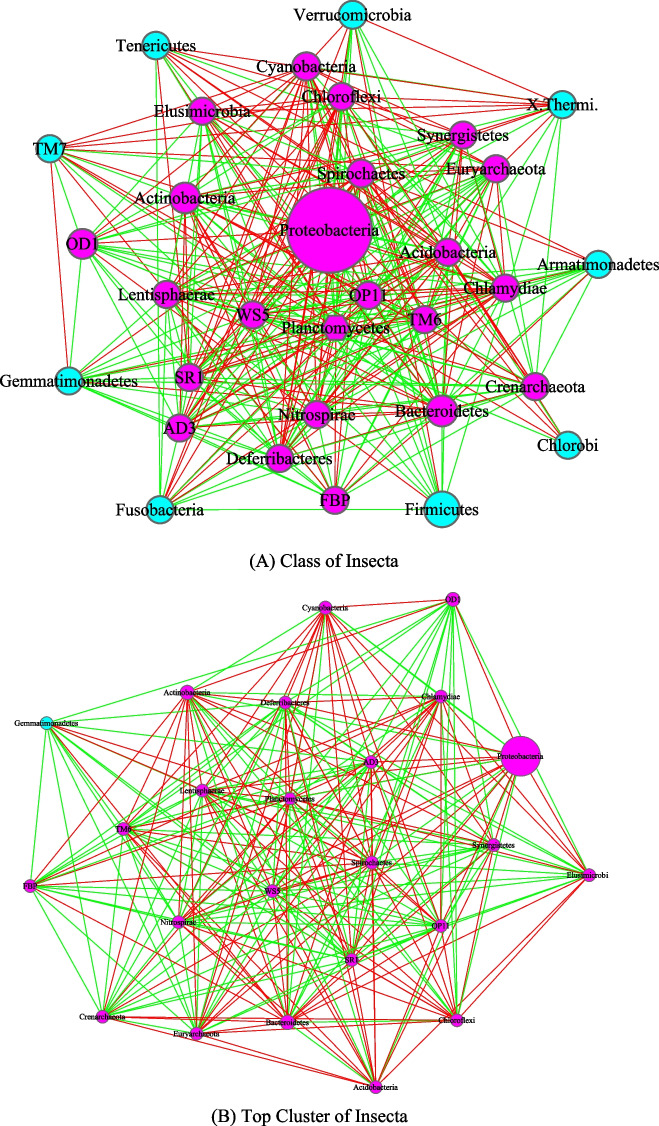

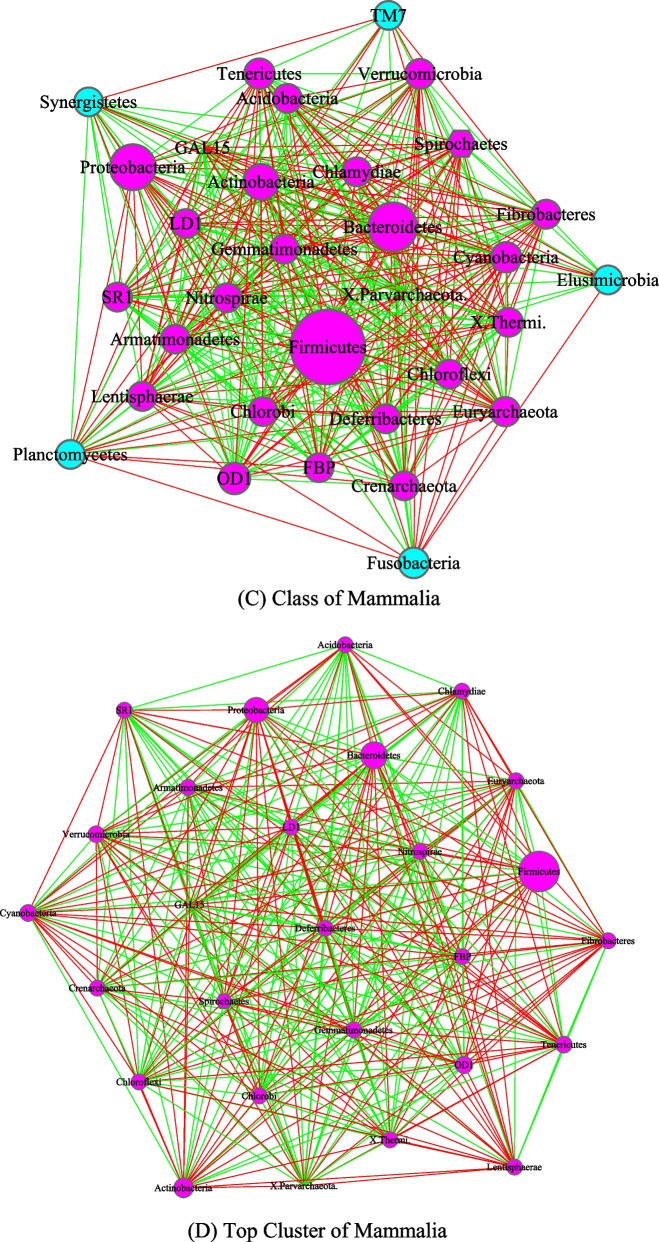
The core/periphery networks (CPN) of the class Insecta and class Mammalia, as well as their top clusters: **A** Class of Insecta; **B** top cluster of Insecta; **C** Class of Mammalia; **D** top cluster of Mammalia. Legends: circle in pink represents for core nodes; circle in cyan for periphery nodes; hexagon for network hub; green line for positive correlation; red line for negative correlation

**Fig. 5:**
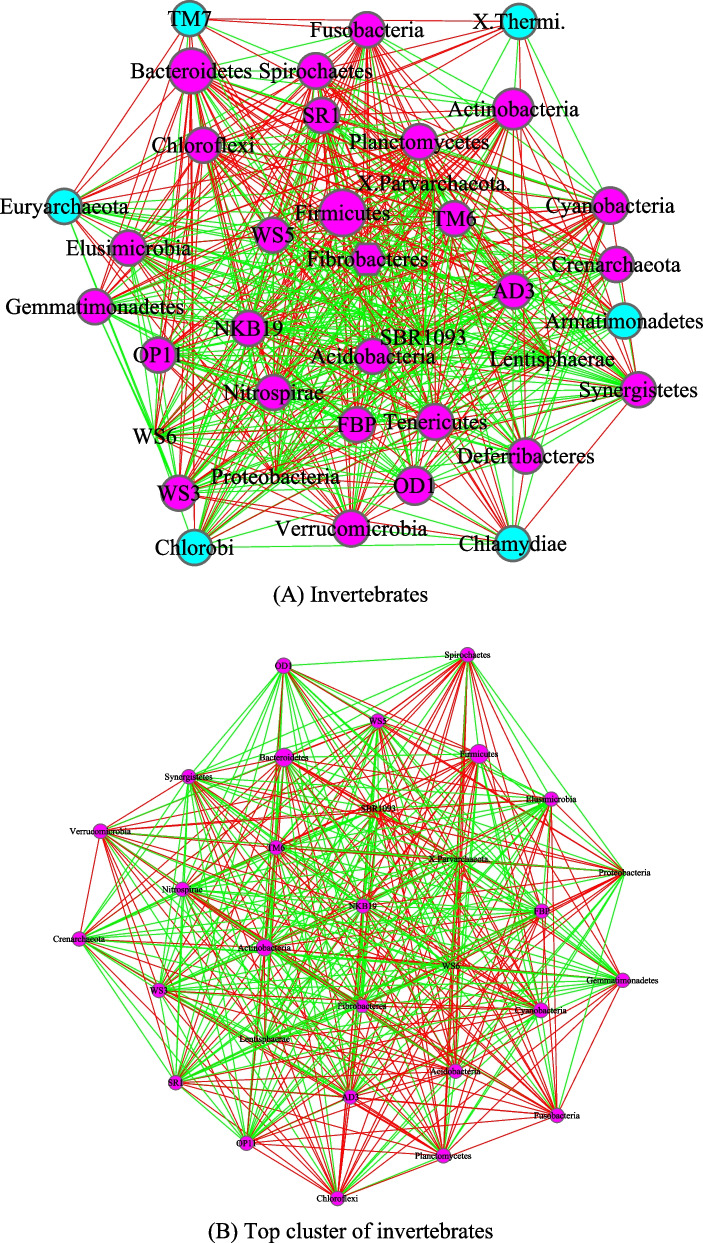

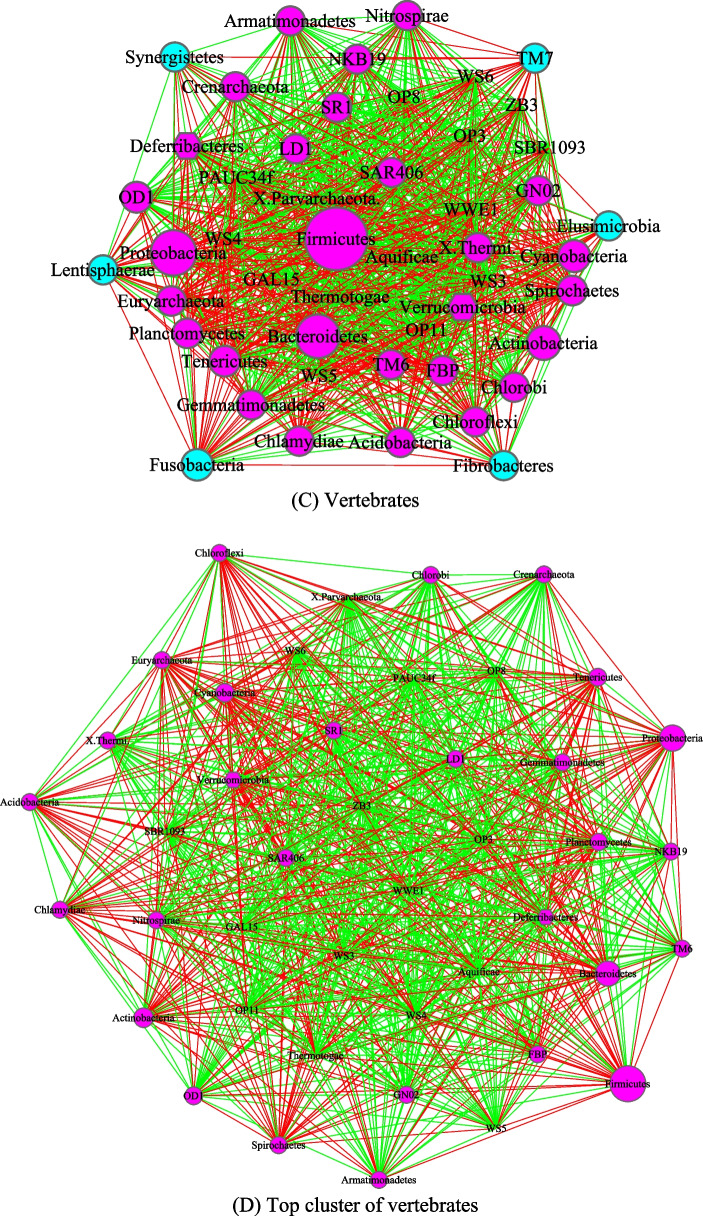
The core/periphery networks (CPN) of Invertebrates and Vertebrates, as well as their top clusters: **A** Invertebrates; **B** top cluster of Invertebrates; **C** vertebrates; **D** top cluster of Vertebrates. Legends: circle in pink represents for core nodes; circle in cyan for periphery nodes; hexagon for network hub; green line for positive correlation; red line for negative correlation

**Fig. 6:**
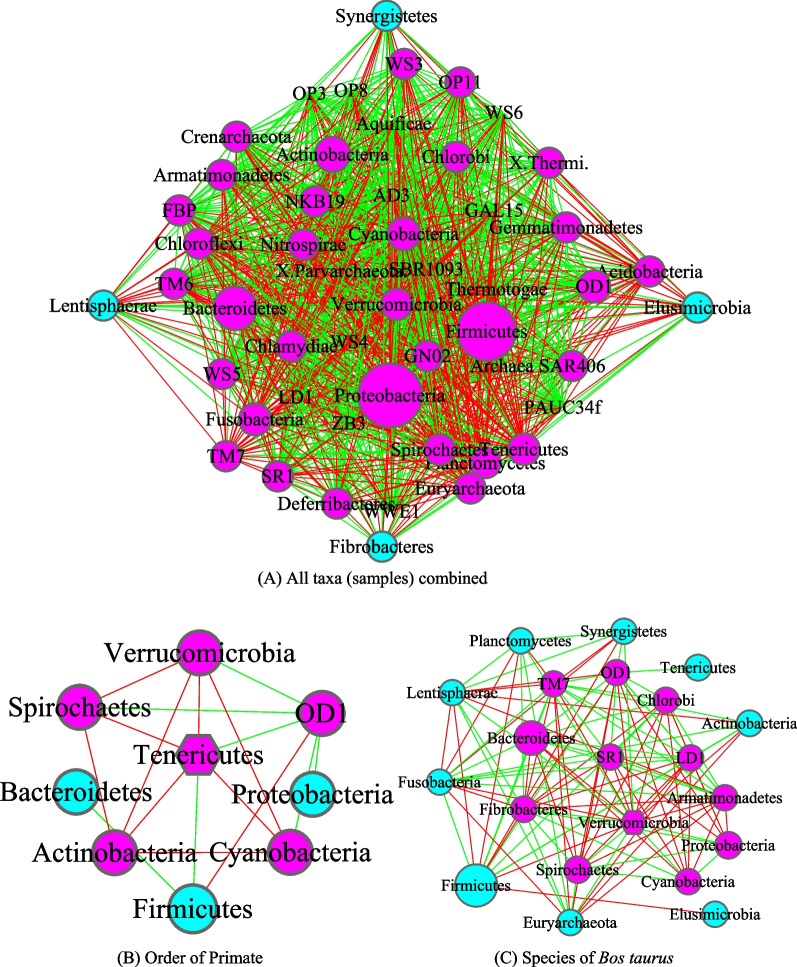
The core/periphery networks (CPN) of three taxa: **A** All taxa (samples) combined; **B** Oder of Primate; **C** Species (*Bos taurus*). Legends: circle in pink represents for core nodes; circle in cyan for periphery nodes; hexagon for network hub; green line for positive correlation; red line for negative correlation

Figures 3, 4, 5 and 6 are the network graphs of the selected AGM networks, whose CPN/HSN properties were exhibited in Additional file 1: Table S1-S6. In these network graphs, the core nodes and periphery nodes are represented in ping and cyan circles, respectively. The network hubs are represented with hexagons. Positive and negative network links (correlations) are represented with green and red lines, respectively. The size of the circles (network nodes) are proportional to the OTU abundances. In each of the figures, except for the last Fig. 6, each network graph is accompanied by a sub-network extracted from the “mother” network, i.e., its strongest module (cluster) detected with MCODE software package. Since the microbial species level networks are overly dense and difficult to display, the selected network graphs in the main article (Figs. 3, 4, 5, 6) are AGM networks were built at microbial phylum level, which are more suitable for visually exploring the network structural features. Although there can be multiple modules (clusters) in a complex network, there is only one strongest module detected in virtually all phylum level AGM networks we constructed. That is, a single module covers virtually all strongly connected nodes in an AGM network at the phylum level. Furthermore, the module contains almost all of the code nodes and occasionally a few peripheral nodes. In some cases, the strongest cluster simply consists of the core nodes. This phenomenon simply cross-confirms the strong core/periphery structures of the AGM networks. In other words, the CPN analysis and module detections with MCODE software generated consistent, mutually cross-verifying results.

Figure 3 illustrated the network graphs of three diet types, i.e., the three AGM networks built with the microbiome samples collected from carnivores, herbivores, and omnivores respectively. Each of the three networks is accompanied by its top cluster, and hence a total of six network graphs are included in Fig. 3. The clusters are ordered by their scores from MCODE software package. It is obvious that BFP (*Bacteroidetes, Firmicutes* and *Proteobacteria*), with their significantly large circle sizes, are the main taxa in the AGM networks. Detailed information on the BFP and their ratios is referred to Additional file 1: Table S6, as explained in the previous sub-section.

Figure 4 was drawn to compare the AGM networks of Insecta and Mammalia class, as well as their top clusters, and Fig. 5 to compare the networks of invertebrates, including their top clusters. Figure 6 includes three sub-graphs, corresponding to the AGM networks of (1) all taxa (samples) combined, (2) order of *Primate*, and (3) a selected species (*Bos taurus*).

The core/periphery network structures, consisting of densely connected core nodes and scattered periphery nodes that are loosely linked to the core, are rather obvious in the network graphs shown in Figs. 3, 4, 5 and 6. The core nodes (Additional file 1: Table S3) and high-salience skeletons (links with high salience values) (Additional file 1: Table S4) constitutes the critical network structures and they should play important roles in maintaining the functions and stability of the animal gut microbiomes. Nevertheless, our current understanding of those functional and stability implications is rather limited. The information is particularly scarce if the context is limited to the animal hosts since much of the microbiome research has been focused on human microbiomes. Additional file 2: Table S7A-S7B summarized some basic biological information on the MAOs (most abundant OTUs) in the AGM networks built at both microbial species and phylum levels. Additional file 2: Table S7C-S7D summarized some basic information on the network hubs (the nodes with the highest links) in the AGM networks at both microbial species and phylum levels. All of the information summarized are from existing literatures and citations are included in Additional file 2: Table S7A-S7D. Obviously, the MAO and hub are likely of rather significant importance for the structure and functions of AGM networks.

The module detection with MCODE software generated the strongest clusters in terms of the cluster scores, which are likely of particular importance. Most of the 14 selected AGM networks were designed (selected) to compare important taxa (e.g., *Insecta* vs. *Mammalia*; *Invertebrates *vs. *Vertebrates*) or diet types (herbivores vs. omnivores, carnivores vs. herbivores, carnivores vs. omnivores). For each comparison, if we compare two networks directly, the workload would be too extensive. For this, we compared their top clusters, and compiled unique and shared OTU lists for each cluster. Additional file 2: Table S8A and S8B summarized some basic biological insights on those cluster-specific unique phyla OTUs. For shared (common) phyla between the top clusters of two taxa or diet types, besides summarizing interesting biological insights, we also compute the abundance ratios of shared species to provide a rough gauge on the direction (increase or decrease) of the shared species, whether it is enriched or impoverished in a particular taxa.

## Conclusions and discussion

From previous sections, we summarized the following findings:

1. The AGM (animal gastrointestinal microbiome) networks follow typical core/periphery nested structures from the network node perspective and contain high-salience skeleton paths from the network link perspectives. That is, all node/links in the AGM network are not homogenous; instead, both nodes and links are heterogenous with differentiated importance. The CPS structures are likely to play major roles in maintaining the functionalities of the AGM networks, and non-critical structures (periphery nodes and low-salience links) are likely to play important roles in stabilizing the networks by offering the network redundancy. In addition, the core/backbone should be like housekeeping genes in genetic networks, being general and ubiquitous.
2. Host phylogeny measured in phylogenetic timeline (PT) does not seem to have significant influences on the evolution of CPS network properties, and our interpretation for the lack of consistent evolutionary patterns in CPS parameters is that the complex networks capture the ecological interactions on ecological time scales, which may fail to emerge in the PT-network parameters models we could reconstruct. The CPS networks of different diet types seem to differ, but we could not determine their statistical significance. While we could not relate holistic network parameters such as core strengthen to host phylogeny, we successfully detected some interesting microbial phylum level relationships with host phylogeny and diet types, especially three primary phyla in animal and human gut microbiomes, as summarized below.
3. The relationships between the abundances of three primary phyla (BFP or *Bacteroidetes, Firmicutes* and *Proteobacteria*) and die types seem to be consistent with the findings in the human gut microbiomes [29, 30]. The B/F ratio of herbivores (B/F = 0.416) is indeed the highest, followed by omnivores (B/F = 0.325) and carnivores (B/F = 0.266). The B/P exhibited the same consistent trend with B/F and that of the human gut microbiome. That is, herbivorous animals and vegetarians should have higher B/F (B/P) ratios than carnivores and meatarians. The ratio of FP is also consistent with the previous two ratios (B/F, B/P). That is, herbivorous animals and vegetarians should have higher BF/BP/FP ratios than carnivores and meatarians, while omnivores sit between them and behind herbivores.
4. The evolution trends of the three key phyla (i.e., BFP) in the animal microbiomes, and especially the evolution of their ratios do not seem to be fully consistent with the patterns found in modern human populations, especially in meatarians or obese populations. Our analyses suggested that both B & F abundances appear to be negatively correlated with PT, and P abundance is positively correlated with PT, implying that more recent (modern) species should have higher B & F abundances, lower P abundance than ancient species, although the correlations between PT with B & F may not be statistically significant (the correlation with P is statistically marginally significant, *P* value = 0.079). Furthermore, the three ratios B/F, B/P, and FP are negatively correlated with PT, suggesting that more recent species should have higher ratios, although the relationship between B/F ratio and PT may be statistically insignificant. Therefore, if the evolutionary trend in animals is to continue in humans, then we should have higher B/P and F/P ratios, rather than lower ratios as exhibited by some obese populations [29, 30].
5. Regarding pairwise correlations between B, F and P, the correlation between B and F is not statistically significant, that between B and P is statistically marginally significant (*P* value = 0.097), but the *negative* correlation between F & P is indeed significant statistically (*P* < 0.005). That is, the phyla of F and P are inhibitory with each other. Combined with the previous findings that the relationship between the abundance of phylum P and its PT is positively correlated, while the relationships of the other two phyla (B & F) and their PT were not statistically significant, we postulate that the evolution of phylum *P* among animals may have more far-reaching influences on the evolution of BFP ratios, than the phyla B & F per se may have on BFP ratios. In other words, more attention to P is deserved than to B & F, to deepen our understanding of the evolution of BFP and their ratios. In existing studies on the human microbiome, much attention has been on B/F or B/P, and little attention has been paid to F/P ratio, which is negatively correlated as this study has suggested.
6. We further cross-verify and supplemented the CPS network analyses with the module detection technique based on MCODE algorithm that detect closely linked clusters. In general, the MCODE algorithm can detect multiple (usually at least 3–5 strong clusters or modules). In the case of microbial phylum-level AGM networks in this study, only one strong cluster was detected in virtually all networks we selected to construct. Furthermore, predominantly majority of the nodes in the strong clusters from MCODE were core nodes of the CPN networks. This confirms the robustness of the CPS analyses and high reliability of the CPS findings. Finally, we summarized, from existing literature, some biological information on the critical taxa (phyla, genera, species) in the critical CPS structures, such as the MAO (most abundant OTU), network hub, host-taxon specific unique or shared OTUs in critical network structures. Nevertheless, due to the current information scarcity on specific microbial OTUs in animal microbiomes, the information we summarized (Additional file 2: Table S7 and S8) is mainly from literatures of human and environmental microbiomes, animal specific information is rather limited in our summary, which calls for more future studies on animal microbiomes.

Cordero and Datta [12] argued that microbial species may co-aggregate for mutual benefits and may segregate to alleviate the effects of competitions. The balance between co-aggregation and segregation can establish distinct local microbial communities and regional metacommunities at larger scales through dispersal/migration. To fully understand the roles of species interactions may play in driving community functionalities, it is imperative to investigate the spatial distribution (organization or structure) with sufficient “resolution” or throughput to measure statistical correlations between taxa and possible alternative community states. We fully agree with Cordero and Datta [12] arguments and adopted a hierarchical design, from both animal host and microbial taxa perspectives besides diet types, to build and analyses the CPS networks.

Vellend [69] proposed to synthesize the community ecology, similar to the synthesis of population genetics, based on the four ecological/evolutionary processes including local selection, local speciation/extinction, global dispersal (migration) and random drifts. In the context of animal or human microbiomes, Näpflin and Schmid-Hempel [52] identified two open questions: First, are there most protective microbiomes to hosts? Second, how much influences does the host exert on shaping the composition and structure of its microbiome? In other words, understanding the bidirectional interactions between animal hosts and their symbiotic microbiomes should be the key for animal microbiome research. Host phylogeny and diet types are arguably the top two most important host factors for animal microbiome research. The phylogenetic timeline (PT, also known as evolutionary timeline), which is different from commonly used PD (phylogenetic distance) and can be considered as an approximate “evolutionary age” of an animal taxon. The more recent species should have smaller PT value and be “young” in terms of the evolutionary “age”. For example, the human has a PT value of 0.6 according to http://timetree.org. The usage of PT information allowed us investigating not only the possible influences of host phylogeny on the CPS structures, but also the evolutionary trend of key AGM taxa. This enabled us to obtain important comparative insights on the BF (*Bacteroidetes*/*Firmicutes*), BP (*Bacteroidetes*/*Proteobacteria*) ratios in animal and humans, which has been a focus of studies on the relationship between human gut microbiomes and personalized nutrition [30].

Existing studies on the relationships between human gut microbiomes and host lifestyles have suggested that modern urban lifestyles (such as eating more high-saturated fat and lower-fiber diets) in the Anthropocene Epoch, especially after industrial revolution, may have led to the rise of *Firmicutes* and *Proteobacteria* and the decline of *Bacteroidetes* abundances in the human gut microbiome (e.g., [29, 30]). Our analysis here is aimed to discover whether or not the trend in the human gut microbiome may have certain traces in the evolution of animal gut microbiomes.

In perspective, our study suggests that the evolutionary trends of B/F and B/P ratios in animal microbiomes, from phylogeny perspective, do not mirror the observed patterns in modern human populations, especially in obese populations dieting on more high-saturated fat and lower-fiber foods [30]. This seems to suggest that the high BF/BP ratios in obese populations should be due to “artificial selection”, rather than natural selection. Our study also calls for more attention on the antagonistic relationships between *Firmicutes* and *Proteobacteria* or the F/P ratio, which is currently paid relatively little attention. From host diet type perspective, our finding from AGM networks seems to be consistent with the finding from humans—herbivores and vegetarians do exhibit highest B/F and B/P ratios, followed by carnivores and omnivores. Additional file 1: Table S6A also listed the BFP ratios for a human population with B/F = 0.286, which is slightly smaller than carnivores (B/F = 0.325) but slightly higher than omnivores (B/F = 0.266) and seems to be a reasonable estimate for humans. The B/P (1.484) and F/P (5.179) for this human population are much larger than those for animals, which is puzzling. Another study on Ukrainian population suggested a B/F range between 0.63 and 1.42 depending on BMI index, age, gender, physic activity, and cigarette smoking [29]. Obviously, comparison with human data is difficult and should be treated with caution. Still from these comparative studies, we postulate that the evolution of the critical compositional phyla (*Bacteroidetes, Firmicutes* and *Proteobacteria*) from animals to humans may have broken the trend, which highlights the far-reaching influences of agriculture and industrial revolution on the human gut microbiomes. In other words, the balances among *Bacteroidetes, Firmicutes* and *Proteobacteria* in the human gut microbiomes in the Anthropocene epoch have been deviating from those of our hunter-gather ancestors and animals.

Finally, we should note a limitation of this study. As introduced in the material and methods section, our study reanalyzed the AGM datasets from 108 published studies containing 6900 AGM samples covering 5 phyla and 19 classes of the animal kingdom. To deal with potential heterogeneities across the different studies, we implemented strict quality control, and only selected 4903 samples covering three primary animal phyla (*Nematoda*, *Arthropoda* and *Chordates*), 10 classes (including all six vertebrate classes and four major invertebrate classes), 59 orders, 142 families, 261 genera, and 318 animal species. To further minimize the influences of potential heterogeneities across the different studies, we recalculated the OTU tables with same bioinformatics pipelines and standard parameters from the original sequencing reads of the respective studies. We also designed and performed rigorous randomization tests whenever computationally feasible to differentiate treatment effects (the influences of phylogeny and diet types) from random noises. Despite these efforts, the results (findings) generated from our analyses may still be subject to possible influences from the heterogeneities of different studies we relied on. Indeed, dealing with the heterogeneity is a rather challenging problem, and we will be conducting additional heterogeneity investigation with these datasets from different perspectives.

### Supplementary Information

**Additional file 1**. Supplementary Tables (Computational Results).

**Additional file 2**. Supplementary Background Information on Special Animal Microbes.

## Data Availability

All data reanalyzed in this study are available in public domain. A summary information can be found in Ma ZS, WD Li & P Shi (2022) Microbiome–host-phylogeny relationships in animal gastrointestinal tract microbiomes, FEMS Microbiology Ecology, Vol. 98(2), fiac021, https://doi.org/10.1093/femsec/fiac021.

## Abbreviations

AGM: Animal gastrointestinal tract microbiomes
CPN: Core/periphery network
HSN: High-salience skeleton network
ET/PT: Evolutionary (phylogenetic) timeline
CPS: Core/periphery/skeleton structures
BF: *Bacteroidetes*/*Firmicutes* (B/F) ratio
BP: *Bacteroidetes*/*Proteobacteria* (B/P) ratio
FP: *Firmicutes*/*Proteobacteria* (F/P) ratio
