# Supplementary material for: Critical complex network structures in animal gastrointestinal tract microbiomes
Source: Anim Microbiome. 2024 May 3;6:23. doi: 10.1186/s42523-024-00291-x (PMC11067214; doi:10.1186/s42523-024-00291-x)
Supplement: Supplementary file 1 — Additional file 1. Supplementary Tables (Computational Results). [file 42523_2024_291_MOESM1_ESM.pdf]

Online Supplementary Information (OSI) for "Critical complex network structures in the animal gastrointestinal tract microbiomes"

Supplementary Tables 1-6

Table S1A. The basic sample/network information for the 14 selected AGM (animal gastrointestinal-tract microbiome) networks of various animal taxa or die types and for the 22 selected AGM networks of 22 animal orders, both at the microbial species level (the OTU or species correlations were computed with SparCC algorithm with FDR control of *P*-value=0.05).

Taxon or Diet Types	Sub-Taxon	Number of Samples	Number of Links	Number of Nodes	Network Density	Positive Links (+)	Negative Links (-)	P/N (+/-) Ratio
*The 14 selected AGM networks of various animal taxon levels and diet types at microbial species level**								
Class	*Chromadorea*	216	12004	991	12.113	11439	565	20.246
	Insecta	979	21527	372	57.868	9406	12121	0.776
	Actinopteri	1271	88611	696	127.315	34564	54047	0.640
	Sauropsida	308	74560	1762	42.316	61104	13456	4.541
	Aves	503	55951	1002	55.839	41547	14404	2.884
	Mammalia	1499	49237	403	122.176	18794	30443	0.617
Diet types	*Carnivore*	1474	69389	593	117.013	25919	43470	0.596
	Herbivore	1621	35874	346	103.682	11761	24113	0.488
	Omnivore	1505	74095	564	131.374	29317	44778	0.655
Vertebrates *vs*. Invertebrates	*Invertebrates*	1269	16036	289	55.488	5783	10253	0.564
	Vertebrates	3634	35634	313	113.847	16367	19267	0.849
Species	*Apis mellifera**	231	1113	135	8.244	660	453	1.457
	*Bos Taurus**	105	40397	335	120.588	19172	21225	0.903
All combined	*All**	4904	93794	836	112.194	34274	59520	0.576
Mean		1394.214	47730.143	616.929	84.290	22864.786	24865.357	2.557
Str.Err.		363.114	7854.560	113.579	11.723	4335.842	5067.377	1.395
*,** Except for the noted networks with stars (*) (representative species & "all combined" networks, for which the threshold of 5% and 1% were adopted, respectively), the threshold of 2% was used to build the other 11 networks at the top section. That is, the OTUs with occurrence frequency across all samples of a treatment below the threshold were excluded from the network constructions.								
The AGM networks of 22 animal orders at microbial species level#								
Chromadorea	*Rhabditida*	215	22729	328	69.296	10747	11982	0.897
Malacostraca	*Amphipoda*	10	1912	529	3.614	1912	0	Inf
Insecta	*Blattodea*	53	157559	1218	129.359	82817	74742	1.108
	Diptera	103	13627	432	31.544	8448	5179	1.631
	Hymenoptera	449	22999	289	79.581	10709	12290	0.871
	Lepidoptera	197	210	48	4.375	86	124	0.694
	Orthoptera	54	2486	236	10.534	2153	333	6.465
Actinopteri	*Cypriniformes*	252	23117	364	63.508	10151	12966	0.783
	Salmoniformes	597	35103	348	100.871	17654	17449	1.012
	Cichliformes	152	50568	831	60.852	32033	18535	1.728
Sauropsida	*Squamata*	284	91694	939	97.651	51868	39826	1.302
Aves	*Anseriformes*	22	1443	328	4.399	1377	66	20.864
	Columbiformes	63	2059	356	5.784	1927	132	14.598
	Passeriformes	80	6956	371	18.749	3758	3198	1.175
	Psittaciformes	48	1404	144	9.750	690	714	0.966

	Struthioniformes	171	8182	746	10.968	6877	1305	5.270
Mammalia	*Carnivora*	382	32213	343	93.915	13802	18411	0.750
	Chiroptera	53	1870	276	6.775	1444	426	3.390
	Cingulata	35	3443	470	7.326	3165	278	11.385
	Diprotodontia	50	56091	423	132.603	26923	29168	0.923
	Primates	234	74698	781	95.644	38060	36638	1.039
	Rodentia	67	1369	259	5.286	1335	34	39.265
Mean		162.318	27806.000	457.227	47.381	14906.182	12899.818	5.529
Str.Err.		32.937	8282.381	59.682	9.688	4378.378	3946.080	2.009

\# *The* 5% threshold of occurrence frequency was adopted to filter out spurious rare species for the animal-order AGM networks.

Table S1B. The basic sample/network information for the 8 selected AGM networks of various animal taxa and diet types at microbial phylum level (the OTU or phylum correlations were computed with SparCC algorithm with FDR control of *P*-value=0.05).

**Taxon or Diet Types**	**Sub-taxon**	**Num. of Samples**	**Number of Links**	**Number of Nodes**	**Network Density**	**Positive Links (+)**	**Negative Links (-)**	**P/N (+/-) Ratio**
Class	*Chromadorea*	216	68	30	2.267	39	29	1.345
	Insecta	979	323	32	10.094	171	152	1.125
	Actinopteri	1271	719	54	13.315	401	318	1.261
	Sauropsida	308	164	31	5.290	85	79	1.076
	Aves	503	250	36	6.944	126	124	1.016
	Mammalia	1499	411	32	12.844	206	205	1.005
Diet types	*Carnivore*	1474	525	40	13.125	289	236	1.225
	Herbivore	1621	582	37	15.730	288	294	0.98
	Omnivore	1505	713	42	16.976	395	318	1.242
Vertebrates *vs*. Invertebrates	*Invertebrates*	1269	543	38	14.289	288	255	1.129
	Vertebrates	3634	1013	49	20.673	549	464	1.183
Species	*Apis mellifera*	231	*NA*	0	*NA*	*NA*	*NA*	*NA*
	Bos taurus	105	117	21	5.571	61	56	1.089
All combined	*All**	4904	1118	58	19.276	616	502	1.227
Mean		1483.692	503.538	38.462	12.030	270.308	233.231	1.146
Str.Err		366.287	87.811	2.753	1.516	48.627	39.360	0.030

Table S2A. The core/periphery network properties for the 14 selected AGM networks, and the 22 selected AGM networks of 22 *animal orders*, both at the microbial species levels

Taxon or Diet Types	Sub-Taxon	*ρ*	Ratio of C/(C+P)	Density Matrix			P/N Ratio				Nestedness (*S*)
				B11	B12(21)	B22	Whole	Core	Peri.	C-P	
The 14 selected AGM networks of the various taxa and diet types											
Class	*Chromadorea*	0.122	0.511	0.073	0.026	0.013	20.246	16.698	153.143	22.221	0.087
	Insecta	0.272	0.465	0.552	0.308	0.137	0.776	0.654	5.072	0.582	0.658
	Actinopteri	0.210	0.543	0.523	0.350	0.185	0.640	0.562	1.405	0.597	0.727
	Sauropsida	0.089	0.499	0.088	0.048	0.028	4.541	3.631	9.090	4.799	0.152
	Aves	0.105	0.528	0.143	0.085	0.052	2.884	1.979	11.781	3.299	0.240
	Mammalia	0.294	0.533	0.380	0.641	0.831	0.617	0.878	0.680	0.514	0.851
Diet types	*Carnivore*	0.215	0.567	0.243	0.426	0.577	0.596	1.196	0.606	0.445	0.749
	Herbivore	0.338	0.558	0.848	0.569	0.290	0.488	0.493	0.943	0.416	0.883
	Omnivore	0.256	0.502	0.687	0.460	0.256	0.655	0.657	1.061	0.568	0.826
Vertebrates *vs*. Invertebrates	*Invertebrates*	0.285	0.516	0.617	0.385	0.124	0.564	0.598	3.779	0.397	0.764
	Vertebrates	0.264	0.546	0.550	0.761	0.916	0.849	1.162	0.913	0.714	0.884
Species	*Apis mellifera*	0.243	0.496	0.263	0.052	0.129	1.457	1.230	2.419	1.236	0.158
	Bos taurus	0.309	0.518	0.498	0.739	0.963	0.903	1.048	0.964	0.823	0.903
All combined	*All*	0.189	0.524	0.132	0.279	0.411	0.576	1.460	0.486	0.496	0.772
Mean		0.228	0.522	0.400	0.366	0.351	2.557	2.303	13.739	2.651	0.618
Std. Err.		0.021	0.007	0.066	0.068	0.090	1.395	1.128	10.763	1.544	0.083
The 22 AGM networks of 22 animal orders from 7 animal classes											
Chromadorea	*Rhabditida*	0.285	0.616	0.243	0.486	0.689	0.897	2.070	0.780	0.688	0.668
Malacostraca	*Amphipoda*	0.104	0.480	0.036	0.007	0.007	-1.000	-1.000	-1.000	*NA*	0.023
Insecta	*Blattodea*	0.160	0.516	0.321	0.196	0.124	1.108	1.005	1.393	1.129	0.356
	Diptera	0.145	0.579	0.074	0.151	0.269	1.631	3.902	1.715	1.240	0.277
	Hymenoptera	0.342	0.585	0.316	0.595	0.903	0.871	1.193	1.253	0.634	0.806
	Lepidoptera	0.562	0.375	0.739	0.133	0.057	0.694	0.507	-1.000	0.469	0.425
	Orthoptera	0.258	0.458	0.233	0.053	0.051	6.465	5.183	-1.000	5.322	0.168
Actinopteri	*Cypriniformes*	0.217	0.500	0.530	0.339	0.191	0.783	0.643	2.512	0.664	0.550
	Salmoniformes	0.283	0.543	0.365	0.575	0.903	1.012	1.018	1.030	0.997	0.817
	Cichliformes	0.149	0.503	0.237	0.131	0.085	1.728	1.341	3.515	1.798	0.281
Sauropsida	*Squamata*	0.136	0.517	0.117	0.200	0.331	1.302	2.042	1.185	1.226	0.436
Aves	*Anseriformes*	0.143	0.473	0.070	0.015	0.014	20.864	11.697	-1.000	*NA*	0.040
	Columbiformes	0.147	0.472	0.081	0.022	0.013	14.598	10.763	54.750	21.419	0.061
	Passeriformes	0.330	0.501	0.274	0.057	0.018	1.175	1.080	3.286	1.252	0.224
	Psittaciformes	0.430	0.417	0.460	0.080	0.054	0.966	0.915	2.760	0.682	0.284
	Struthioniformes	0.076	0.524	0.050	0.024	0.016	5.270	4.372	12.547	5.453	0.067
Mammalia	*Carnivora*	0.305	0.574	0.333	0.584	0.850	0.750	1.079	0.831	0.613	0.775
	Chiroptera	0.267	0.402	0.181	0.026	0.021	3.390	2.908	4.088	4.512	0.093
	Cingulata	0.101	0.513	0.061	0.024	0.014	11.385	7.693	60.833	17.800	0.065
	Diprotodontia	0.307	0.527	0.868	0.608	0.376	0.923	0.935	1.461	0.805	0.856
	Primates	0.173	0.525	0.366	0.226	0.141	1.039	0.952	1.479	1.018	0.436

	Rodentia	0.171	0.529	0.096	0.019	0.021	39.265	31.963	154.000	53.000	0.063
Mean		0.231	0.506	0.275	0.207	0.234	5.233	4.194	13.883	5.396	0.353
Std. Err.		0.025	0.013	0.047	0.047	0.066	1.983	1.498	7.560	2.565	0.060

Table S2B. The core/periphery network properties of 9 selected AGM networks of various animal taxa and diet types at microbial phylum level

**Group**	**Classification**	***ρ***	**Ratio of C/(C+P)**	**Density Matrix**			**P/N Ratio**				**Nestedness (*S*)**
				B11	B12(21)	B22	Whole	Core	Peri.	C-P	
Class	*Insecta*	0.510	0.719	0.889	0.401	0.417	1.125	1.027	0.667	1.594	0.796
	Mammalia	0.555	0.844	0.963	0.481	0.800	1.005	0.988	0.600	1.167	0.917
Diet types	*Carnivore*	0.603	0.800	0.887	0.262	0.643	1.225	1.316	0.500	0.971	0.812
	Herbivore	0.493	0.919	0.945	0.480	1.000	0.980	1.023	0.500	0.633	0.936
	Omnivore	0.416	0.429	0.490	0.861	0.964	1.242	1.586	0.970	1.416	0.930
Vertebrates *vs*. Invertebrates	*Invertebrates*	0.468	0.842	0.899	0.474	0.400	1.129	1.124	0.200	1.275	0.887
	Vertebrates	0.450	0.878	0.947	0.566	0.800	1.183	1.388	1.000	0.460	0.925
Species	*Bos taurus*	0.542	0.571	0.955	0.324	0.528	1.089	0.969	0.900	1.500	0.786
Order	*Primate*	0.668	0.667	0.867	0.167	0.333	0.700	0.444	-1.000	2.000	0.696
Mean		0.523	0.741	0.871	0.446	0.654	1.075	1.096	0.482	1.224	0.854
Std. Err.		0.025	0.051	0.046	0.063	0.078	0.052	0.102	0.193	0.151	0.027

Table 3SA. A brief summary on the 22 selected AGM networks of 22 of animal orders at microbial species level

Class	Order	PT (Phylogenetic Timeline)	Num. of OTU	Core-Periphery			Num. of high-Salience Skeletons (Salience value≥0.5)
				Num. of Core Nodes (C)	Num. of Periphery Nodes (P)	Ratio of C/P	
Chromadorea	*Rhabditida*	181	328	202	126	1.603	88
Malacostraca	*Amphipoda*	334	529	254	275	0.924	0
	Blattodea	228	1218	629	589	1.068	124
	Diptera	272	432	250	182	1.374	26
Insecta	*Hymenoptera*	325	289	169	120	1.408	29
	Lepidoptera	232	48	18	30	0.600	28
	Orthoptera	295	236	108	128	0.844	134
	Cypriniformes	150	364	182	182	1.000	48
Actinopteri	*Salmoniformes*	128	348	189	159	1.189	71
	Cichliformes	95	831	418	414	1.010	71
Sauropsida	*Squamata*	252	939	485	454	1.068	52
	Anseriformes	80	328	155	173	0.896	225
	Columbiformes	82	356	168	188	0.894	188
Aves	*Passeriformes*	82	371	186	185	1.005	213
	Psittaciformes	82	144	60	84	0.714	55
	Struthioniformes	90	746	391	355	1.101	260
	Carnivora	75	343	197	146	1.349	75
	Chiroptera	79	276	111	165	0.673	151
Mammalia	*Cingulata*	66	470	241	229	1.052	275
	Diprotodontia	62	423	223	200	1.115	3
	Primates	76	781	410	371	1.105	69
	Rodentia	82	259	137	122	1.123	176
Mean		152.182	457.227	235.591	221.682	1.051	107.318
Std. Err.		20.060	59.682	31.240	28.833	0.052	17.828

Table S3B. A brief summary on the core/periphery and high-salience skeleton networks for the 8 selected AGM networks at the microbial *phylum* levels.

Taxon or Diet Types	Sub-Taxon	Num. of OTU	Core-Periphery					Num. of high-salience skeletons with (Salience value≥0.2)
			Num. of Core Nodes	Num. of Periphery Nodes	Ratio of C/P	Core	Periphery	
Class	*Insecta*	32	23	9	2.556	*Proteobacteria*<br>*OD1*<br>*Planctomycetes*<br>*Bacteroidetes*<br>*Actinobacteria*<br>*Chlamydiae*<br>*Deferribacteres*<br>*Cyanobacteria*<br>*Spirochaetes*<br>*Euryarchaeota*<br>*Acidobacteria*<br>*Chloroflexi*<br>*SR1*<br>*TM6*<br>*FBP*<br>*Nitrospirae*<br>*Elusimicrobia*	*Firmicutes*<br>*Tenericutes*<br>*Fusobacteria*<br>*Verrucomicrobia*<br>*TM7*<br>*Chlorobi*<br>*Armatimonadetes*<br>*Gemmatimonadetes*<br>*X.Thermi.*	52

						OP11<br>*Lentisphaerae*<br>*Crenarchaeota*<br>*Synergistetes*<br>*AD3*<br>*WS5*		
	Mammalia	32	27	5	5.400	*Proteobacteria*<br>*OD1*<br>*Firmicutes*<br>*Bacteroidetes*<br>*Tenericutes*<br>*Actinobacteria*<br>*Chlamydiae*<br>*Deferribacteres*<br>*Cyanobacteria*<br>*Spirochaetes*<br>*Verrucomicrobia*<br>*Euryarchaeota*<br>*Acidobacteria*<br>*Chloroflexi*<br>*Fibrobacteres*<br>*SR1*<br>*Chlorobi*<br>*Armatimonadetes*<br>*Gemmatimonadetes*<br>*X.Thermi.*<br>*X.Parvarchaeota.*<br>*FBP*<br>*Nitrospirae*<br>*Lentisphaerae*<br>*Crenarchaeota*<br>*GAL15*<br>*LD1*	*Planctomycetes*<br>*Fusobacteria*<br>*TM7*<br>*Elusimicrobia*<br>*Synergistetes*	28
Diet types	*Carnivore*	40	32	8	4.00	*Proteobacteria*<br>*Firmicutes*<br>*Planctomycetes*<br>*Bacteroidetes*<br>*Actinobacteria*<br>*Chlamydiae*<br>*Deferribacteres*<br>*Cyanobacteria*<br>*Verrucomicrobia*<br>*Acidobacteria*<br>*Chloroflexi*<br>*WS3*<br>*Fibrobacteres*<br>*SR1*<br>*TM6*<br>*Chlorobi*<br>*OP3*<br>*Armatimonadetes*<br>*Gemmatimonadetes*<br>*X.Parvarchaeota.*<br>*NKB19*<br>*FBP*<br>*Nitrospirae*<br>*SBR193*<br>*Lentisphaerae*<br>*Crenarchaeota*<br>*Thermotogae*<br>*Synergistetes*<br>*SAR46*<br>*ZB3*<br>*PAUC34f*<br>*WS5*	*OD1*<br>*Tenericutes*<br>*Fusobacteria*<br>*Spirochaetes*<br>*Euryarchaeota*<br>*TM7*<br>*X.Thermi.*<br>*Elusimicrobia*	53
	Herbivore	37	34	3	11.333	*Proteobacteria*<br>*OD1*<br>*Firmicutes*<br>*Planctomycetes*<br>*Bacteroidetes*<br>*Tenericutes*<br>*Actinobacteria*<br>*Fusobacteria*<br>*Chlamydiae*<br>*Deferribacteres*<br>*Cyanobacteria*<br>*Spirochaetes*<br>*Verrucomicrobia*<br>*Euryarchaeota*<br>*Acidobacteria*	*TM7*<br>*Elusimicrobia*<br>*Synergistetes*	27

						Chloroflexi<br>*WS3*<br>*Fibrobacteres*<br>*SR1*<br>*TM6*<br>*Chlorobi*<br>*Armatimonadetes*<br>*Gemmatimonadetes*<br>*X.Thermi.*<br>*X.Parvarchaeota.*<br>*FBP*<br>*Nitrospirae*<br>*OP11*<br>*Lentisphaerae*<br>*Crenarchaeota*<br>*WWE1*<br>*LD1*<br>*WS5*<br>*WS6*		
	Omnivore	42	18	24	0.750	*Firmicutes*<br>*Bacteroidetes*<br>*Chlamydiae*<br>*Acidobacteria*<br>*Chloroflexi*<br>*TM7*<br>*Fibrobacteres*<br>*SR1*<br>*TM6*<br>*Chlorobi*<br>*Armatimonadetes*<br>*Gemmatimonadetes*<br>*X.Thermi.*<br>*Nitrospirae*<br>*Elusimicrobia*<br>*Lentisphaerae*<br>*Crenarchaeota*<br>*Synergistetes*	*Proteobacteria*<br>*OD1*<br>*Planctomycetes*<br>*Tenericutes*<br>*Actinobacteria*<br>*Fusobacteria*<br>*Deferribacteres*<br>*Cyanobacteria*<br>*Spirochaetes*<br>*Verrucomicrobia*<br>*Euryarchaeota*<br>*WS3*<br>*GN02*<br>*OP3*<br>*X.Parvarchaeota.*<br>*NKB19*<br>*FBP*<br>*OP11*<br>*SBR1093*<br>*GAL15*<br>*WS4*<br>*SAR406*<br>*WS5*<br>*WS6*	51
Vertebrates *vs*. Invertebrates	*Invertebrates*	38	32	6	5.333	*Proteobacteria*<br>*OD1*<br>*Firmicutes*<br>*Planctomycetes*<br>*Bacteroidetes*<br>*Tenericutes*<br>*Actinobacteria*<br>*Fusobacteria*<br>*Deferribacteres*<br>*Cyanobacteria*<br>*Spirochaetes*<br>*Verrucomicrobia*<br>*Acidobacteria*<br>*Chloroflexi*<br>*WS3*<br>*Fibrobacteres*<br>*SR1*<br>*TM6*<br>*Gemmatimonadetes*<br>*X.Parvarchaeota.*<br>*NKB19*<br>*FBP*<br>*Nitrospirae*<br>*Elusimicrobia*<br>*OP11*<br>*SBR193*<br>*Lentisphaerae*<br>*Crenarchaeota*<br>*Synergistetes*<br>*AD3*<br>*WS5*<br>*WS6*<br>*WS6*	*Chlamydiae*<br>*Euryarchaeota*<br>*TM7*<br>*Chlorobi*<br>*Armatimonadetes*<br>*X.Thermi.*	47
	Vertebrates	49	43	6	7.167	*Proteobacteria*<br>*OD1*<br>*Firmicutes*<br>*Planctomycetes*	*Fusobacteria*<br>*TM7*<br>*Fibrobacteres*<br>*Elusimicrobia*	53

						Bacteroidetes<br>*Tenericutes*<br>*Actinobacteria*<br>*Chlamydiae*<br>*Deferribacteres*<br>*Cyanobacteria*<br>*Spirochaetes*<br>*Verrucomicrobia*<br>*Euryarchaeota*<br>*Acidobacteria*<br>*Chloroflexi*<br>*WS3*<br>*SR1*<br>*TM6*<br>*Chlorobi*<br>*GN2*<br>*OP3*<br>*Armatimonadetes*<br>*Gemmatimonadetes*<br>*X.Thermi.*<br>*X.Parvarchaeota.*<br>*NKB19*<br>*FBP*<br>*Nitrospirae*<br>*OP11*<br>*SBR193*<br>*Crenarchaeota*<br>*WWE1*<br>*Thermotogae*<br>*GAL15*<br>*WS4*<br>*LD1*<br>*SAR46*<br>*ZB3*<br>*PAUC34f*<br>*Aquificae*<br>*WS5*<br>*OP8*<br>*WS6*	*Lentisphaerae*<br>*Synergistetes*	
Species	*Bos taurus*	21	12	9	1.333	*Proteobacteria*<br>*OD1*<br>*Bacteroidetes*<br>*Cyanobacteria*<br>*Spirochaetes*<br>*Verrucomicrobia*<br>*TM7*<br>*Fibrobacteres*<br>*SR1*<br>*Chlorobi*<br>*Armatimonadetes*<br>*LD1*	*Firmicutes*<br>*Planctomycetes*<br>*Actinobacteria*<br>*Fusobacteria*<br>*Euryarchaeota*<br>*Lentisphaerae*<br>*Tenericutes*<br>*Elusimicrobia*<br>*Synergistetes*	28
Mean		36.375	27.625	8.750	4.734	-	-	42.375
Std. Err.		2.933	3.469	2.297	1.217	-	-	4.359

Table S4A. The HSN (high-salience skeleton networks) properties of the 14 selected AGM of various animal taxa and diet types and of the 22 animal-order-level AGM networks, both at the microbial species level

Group	Classification	Statistics of HSS							Assortativity
		**Links (%)**	**Max**	**Mean**	**Median**	*Std. Err*	Skewness	Kurtosis	r_{HSS}
The 14 selected AGM-HSN for various taxa and diet types									
Class	*Chromadorea*	5.674	0.988	0.002	0.002	0.034	21.789	533.998	-0.001
	Insecta	16.572	0.981	0.005	0.005	0.036	13.521	229.183	-0.003
	Actinopteri	14.389	0.999	0.003	0.004	0.032	19.816	437.442	-0.001
	Sauropsida	4.440	1.000	0.001	0.004	0.020	32.668	1299.890	-0.001
	Aves	5.126	1.000	0.002	0.004	0.027	23.436	620.290	-0.001
	Mammalia	23.082	0.886	0.005	0.005	0.037	14.073	225.376	-0.002
Diet types	*Carnivore*	15.530	0.998	0.003	0.003	0.037	18.457	370.927	-0.002
	Herbivore	16.736	0.974	0.006	0.006	0.049	13.556	201.153	-0.003
	Omnivore	12.831	0.980	0.004	0.004	0.034	15.333	269.760	-0.002
Vertebrates *vs.* Invertebrates	*Invertebrates*	18.627	0.941	0.007	0.007	0.042	11.477	159.538	-0.003
	Vertebrates	24.334	0.997	0.006	0.006	0.048	13.531	197.376	-0.003
Species	*Apis mellifera*	12.040	1.000	0.015	0.096	0.056	5.445	40.184	-0.007
	Bos taurus	68.718	0.847	0.006	0.006	0.023	23.925	679.596	-0.003
All combined	All	6.931	1.000	0.002	0.002	0.035	20.326	445.655	-0.001
Mean		17.502	0.971	0.005	0.011	0.036	17.668	407.883	-0.002
Std. Err.		4.287	0.013	0.001	0.007	0.003	1.791	84.595	0.000
The 22 AGM-HSN for the 22 animal orders									
Chromadorea	*Rhabditida*	22.652	0.768	0.006	0.009	0.036	11.928	165.280	-0.003
Malacostraca	*Amphipoda*	86.112	0.214	0.004	0.004	0.007	17.405	358.729	-0.002
Insecta	*Blattodea*	21.188	1.000	0.002	0.002	0.014	30.896	1336.735	-0.001
	Diptera	14.297	1.000	0.005	0.014	0.025	12.361	215.876	-0.002
	Hymenoptera	38.886	0.858	0.007	0.007	0.029	13.398	245.425	-0.003
	Lepidoptera	24.202	0.958	0.042	0.063	0.135	4.977	26.860	-0.021
	Orthoptera	8.965	1.000	0.008	0.025	0.060	11.301	144.995	-0.004
Actinopteri	*Cypriniformes*	17.434	0.890	0.005	0.008	0.032	11.664	172.404	-0.003
	Salmoniformes	24.898	0.980	0.006	0.009	0.035	15.802	316.740	-0.003
	Cichliformes	13.821	1.000	0.002	0.006	0.017	21.756	705.137	-0.001
Sauropsida	*Squamata*	15.334	1.000	0.002	0.003	0.016	20.158	628.808	-0.001
Aves	*Anseriformes*	18.429	0.918	0.006	0.006	0.055	13.600	196.208	-0.003
	Columbiformes	10.969	0.961	0.006	0.006	0.050	13.920	221.584	-0.003
	Passeriformes	10.002	1.000	0.005	0.016	0.050	15.997	277.645	-0.003
	Psittaciformes	13.627	1.000	0.014	0.047	0.068	9.296	105.185	-0.007
	Struthioniformes	2.929	1.000	0.003	0.042	0.029	19.395	478.267	-0.001
Mammalia	*Carnivora*	31.848	0.953	0.006	0.006	0.033	15.603	303.015	-0.003
	Chiroptera	24.153	0.899	0.007	0.007	0.054	13.022	185.821	-0.004
	Cingulata	6.916	0.981	0.004	0.004	0.044	15.961	290.112	-0.002
	Diprotodontia	61.205	0.669	0.005	0.005	0.013	19.106	530.441	-0.002
	Primates	15.653	0.880	0.003	0.004	0.019	18.118	441.103	-0.001
	Rodentia	11.598	0.961	0.008	0.008	0.063	11.250	138.254	-0.004
Mean		22.505	0.904	0.007	0.014	0.040	15.314	340.210	-0.004
Std. Err.		4.027	0.038	0.002	0.003	0.006	1.116	59.903	0.001

Table S4B. The HSN (high-salience skeleton network) properties of the 9 selected AGM networks at the microbial phylum level

Group	Classification	Statistics of HSS							Assortativity
		**Links (%)**	**Max**	**Mean**	**Median**	*Std. Err.*	Skewness	Kurtosis	r_{HSS}
Class	*Insecta*	33.871	0.750	0.063	0.063	0.136	2.943	8.714	-0.032
	Mammalia	55.242	0.719	0.063	0.063	0.092	3.040	12.880	-0.032
Diet types	*Carnivore*	35.513	0.825	0.050	0.050	0.118	3.851	16.681	-0.026
	Herbivore	56.306	0.811	0.054	0.054	0.091	4.252	24.189	-0.028
	Omnivore	47.735	0.881	0.048	0.048	0.096	4.319	23.819	-0.024
Vertebrates *vs*. Invertebrates	*Invertebrates*	42.248	0.789	0.053	0.053	0.114	3.695	15.009	-0.027
	Vertebrates	45.323	0.918	0.041	0.041	0.097	4.887	27.771	-0.021
Species	*Bos taurus*	51.429	1.000	0.095	0.143	0.145	3.248	15.491	-0.050
Order	*Primate*	47.222	1.000	0.222	0.444	0.291	1.253	0.932	-0.125
Mean		46.099	0.855	0.077	0.107	0.131	3.499	16.165	-0.041
Std. Err.		2.628	0.034	0.019	0.043	0.021	0.353	2.784	0.011

Table S5. The Spearman's correlation coefficient (*R*) and corresponding *P*-value between Network Density, P/N Ratio, the core-periphery networks (CPN)/high-salience skeleton network (HSN) parameters and phylogenetic timeline (PT) at the *animal-order* level (computed across the PT-values of 22 animal orders): {*However, none of the parameters exhibited significant correlations with the PT as illustrated inside the table*}

**Network Parameter**	**Spearman's Correlation Coefficient (*R*)**	***P*-value**	**Scatter plot**
The basic sample/network information			
Network Density	-0.044	0.843	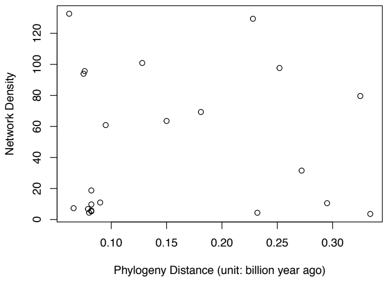
P/N Ratio	-0.182	0.429	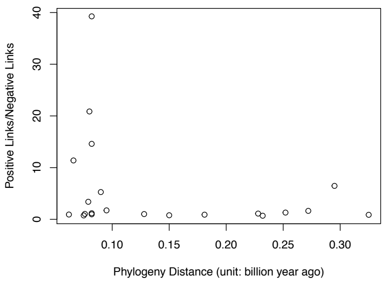
Core/Periphery Networks			
ρ	-0.054	0.810	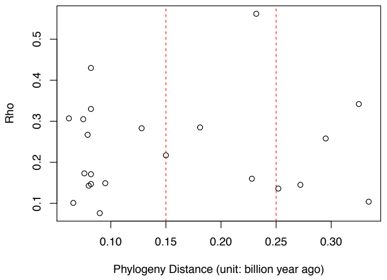

<table>
<tr><td>B11</td><td>-0.139</td><td>0.5364</td><td>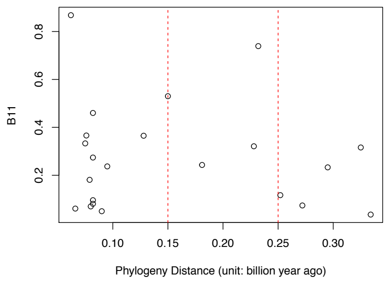
</td></tr>
<tr><td>B12(21)</td><td>0.035</td><td>0.8767</td><td>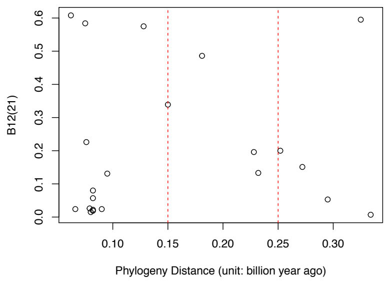
</td></tr>
<tr><td>B22</td><td>0.157</td><td>0.484</td><td>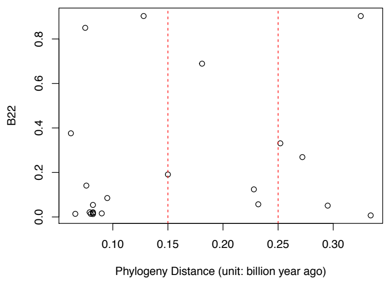
</td></tr>
<tr><td>Nestedness (S)</td><td>0.025</td><td>0.910</td><td>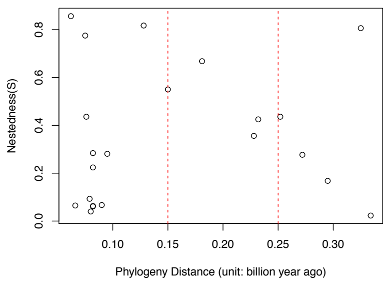
</td></tr>
<tr><td colspan="4">High-salience skeleton networks</td></tr>
</table>

***Links* (%) with Salience>0**	0.140	0.535	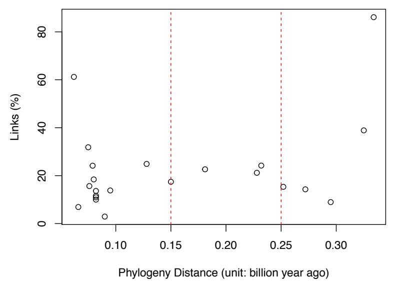
Mean of Salience	0.011	0.960	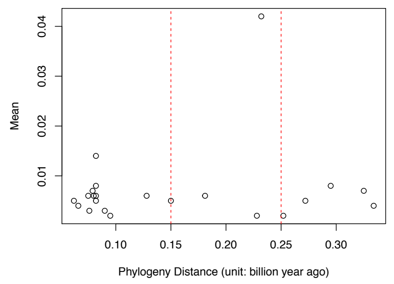
Median of Salience	0.201	0.370	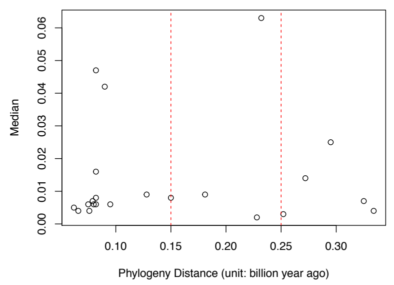

Table S6A. The mean relative abundances and pair-wise ratios for the three phyla *Bacteroidetes*, *Firmicutes* and *Proteobacteria* for the 9 selected AGM networks of various animal taxa (diet types) and the 35 AGM networks at the *animal-order* level (B/F= *Bacteroidetes*/*Firmicutes*; B/P= *Bacteroidetes*/ *Proteobacteria*; F/P= *Firmicutes*/*Proteobacteria*)

Taxon or Diet Types	Sub-Taxon	Bacteroidetes	Firmicutes	Proteobacteria	B/F	B/P	F/P
The 9 selected AGM networks of various animal taxon levels and diet types at microbial phylum level							
Class	*Insecta*	0.032	0.273	0.417	0.116	0.076	0.655
	Mammalia	0.179	0.426	0.199	0.420	0.902	2.146
Diet types	*Carnivore*	0.087	0.326	0.270	0.266	0.321	1.208
	Herbivore	0.161	0.386	0.257	0.416	0.625	1.503
	Omnivore	0.125	0.385	0.273	0.325	0.459	1.413
Vertebrates *vs.* Invertebrates	*Invertebrates*	0.065	0.223	0.472	0.291	0.138	0.473
	Vertebrates	0.148	0.387	0.225	0.382	0.660	1.726
Species	*Apis mellifera*	0.003	0.714	0.223	0.004	0.012	3.195
	Bos taurus	0.438	0.487	0.016	0.900	27.319	30.369
	Homo sapiens	0.160	0.560	0.108	0.286	1.484	5.179
Mean		0.140	0.417	0.246	0.341	3.200	4.787
Std. Err.		0.038	0.045	0.042	0.075	2.684	2.875
The AGM networks of 35 animal orders at microbial phylum level (Computed across all samples of an animal order)							
Chromadorea	*Rhabditida*	0.016	0.000	0.573	Inf	0.029	0.000
Arachnida	*Araneae*	0.008	0.706	0.125	0.012	0.068	5.651
Malacostraca	*Amphipoda*	0.250	0.205	0.250	1.220	1.000	0.820
	Decapoda	0.087	0.802	0.000	0.108	Inf	Inf
Insecta	*Blattodea*	0.063	0.225	0.011	0.280	5.533	19.779
	Coleoptera	0.024	0.423	0.194	0.056	0.123	2.182
	Diptera	0.014	0.187	0.118	0.073	0.117	1.589
	Hemiptera	0.000	0.000	0.000	*NA*	*NA*	*NA*
	Hymenoptera	0.023	0.182	0.407	0.127	0.057	0.446
	Lepidoptera	0.010	0.509	0.413	0.020	0.024	1.233
	Orthoptera	0.069	0.624	0.000	0.110	Inf	Inf
Chondrichthyes	*Carcharhiniformes*	0.042	0.916	0.000	0.046	Inf	Inf
Actinopteri	*Cypriniformes*	0.830	0.146	0.025	5.696	33.742	5.924
	Salmoniformes	0.035	0.085	0.064	0.413	0.554	1.343
	Acanthuriformes	0.333	0.667	0.000	0.500	Inf	Inf
	Centrarchiformes	0.172	0.000	0.695	Inf	0.247	0.000
	Cichliformes	0.006	0.001	0.233	4.497	0.028	0.006
Amphibia	*Anura*	0.000	0.288	0.672	0.000	0.000	0.429
Sauropsida	*Squamata*	0.002	0.156	0.021	0.012	0.088	7.477
Aves	*Anseriformes*	0.495	0.378	0.015	1.308	32.272	24.665
	Charadriiformes	0.042	0.916	0.000	0.046	Inf	Inf
	Columbiformes	0.524	0.085	0.283	6.201	1.849	0.298
	Passeriformes	0.351	0.021	0.600	16.758	0.585	0.035
	Psittaciformes	0.044	0.203	0.000	0.215	Inf	Inf
	Struthioniformes	0.001	0.978	0.005	0.001	0.313	212.072
Mammalia	*Carnivora*	0.045	0.356	0.012	0.125	3.738	29.886
	Chiroptera	*NA*	*NA*	*NA*	*NA*	*NA*	*NA*
	Cingulata	0.049	0.813	0.001	0.061	53.244	875.763
	Diprotodontia	0.036	0.875	0.043	0.041	0.844	20.383
	Perissodactyla	0.071	0.546	0.000	0.130	2321.664	17800.284
	Pilosa	0.195	0.677	0.000	0.288	Inf	Inf
	Primates	0.062	0.811	0.023	0.076	2.717	35.644

	Proboscidea	0.141	0.402	0.070	0.350	2.004	5.725
	Rodentia	0.036	0.605	0.014	0.060	2.582	42.927
	Tubulidentata	0.000	0.792	0.030	0.000	0.000	26.178
Mean		0.120	0.429	0.144	1.253	94.747	735.413
Std. Err.		0.032	0.055	0.037	0.593	89.114	683.438

Table S6B. The Spearman's correlation coefficients between *Bacteroidetes, Bacteroidetes* and *Proteobacteria* of 9 selected AGM networks of various animal taxon levels and diet types and 35 animal orders at microbial phylum level

**Networks**	**Bacteroidetes *vs.* Firmicutes**		**Bacteroidetes *vs.* Proteobacteria**		**Firmicutes *vs.* Proteobacteria**	
	**Spearman's Correlation**	***P*-value**	**Spearman's Correlation**	***P*-value**	**Spearman's Correlation**	***P*-value**
9 selected AGM networks of various animal taxon levels and diet types	0.367	0.336	-0.600	0.097	-0.933	0.001*
35 animal orders	-0.028	0.871	-0.192	0.276	-0.479	0.004*

Fig S6C. The Spearman's correlation coefficients and corresponding *p*-value between the three phyla (*Bacteroidetes*, *Firmicutes* and *Proteobacteria*) and phylogenetic timeline (PT) as well as between the pairwise ratio of the three phyla and PT, computed from the data of 35 animal orders.

**BFP Phylum**	**Spearman's Correlation Coefficient**	***P*-value**	**Scatter plot**
Bacteroidetes	-0.279	0.116	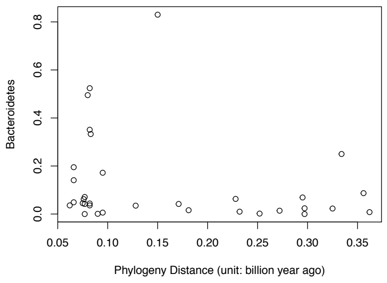
Firmicute	-0.272	0.126	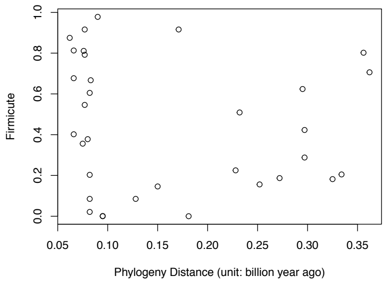

Proteobacteria	0.310	0.079	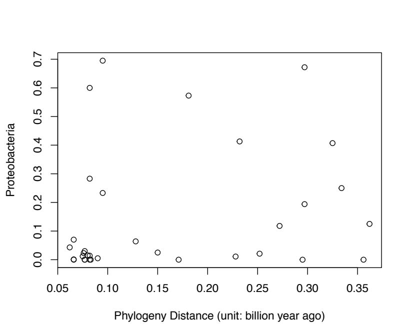
Bacteroidetes/Firmicute	-0.152	0.432	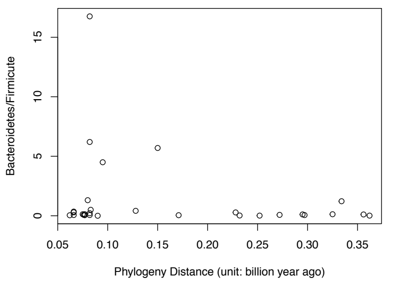
Bacteroidetes/Proteobacteria	-0.569	0.002*	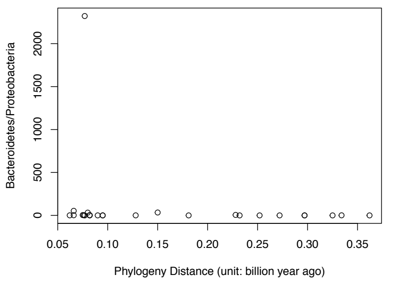
Firmicutes/Proteobacteria	-0.517	0.007*	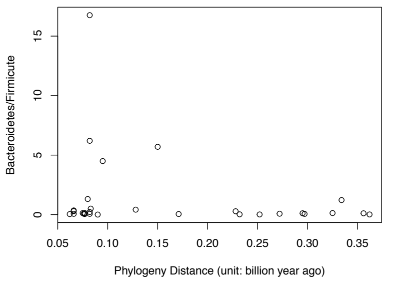
