# Supplementary material for: Critical complex network structures in animal gastrointestinal tract microbiomes
Source: Anim Microbiome. 2024 May 3;6:23. doi: 10.1186/s42523-024-00291-x (PMC11067214; doi:10.1186/s42523-024-00291-x)
Supplement: Supplementary file 2 — Additional file 2. Supplementary Background Information on Special Animal Microbes. [file 42523_2024_291_MOESM2_ESM.pdf]

Online Supplementary Information (OSI) for "Critical network structures in the animal gastrointestinal tract microbiomes"

Table S7A. The basic biological information on the MAOs (most abundance OTUs) for the 14 selected AGM networks and the 22 animal-order AGM networks, both at microbial species levels.

**Host Taxon or Diet Types**	**Animal Sub-Taxon**	**Microbial MAO**	**Description** (most are directly quoted from the sources in the reference column)	**Reference**
The 14 Selected AGM networks for various taxa and diet types				
Class	*Chromadorea*	*Sphingomonas*	"Some species of *Sphingomonas* (especially *S. paucimobilis*) may cause minor infection in humans and damage some other organisms." Quoted from Lombardino et al. (2022)	Lombardino, J., Bijlani, S., Singh, N. K., et al. (2022). Genomic Characterization of Potential Plant Growth-Promoting Features of Sphingomonas Strains Isolated from the International Space Station. *Microbiology Spectrum*, 10(1), e0199421.
	Insecta	*Pasteurellales*	"*Pasteurella pneumotropica* is an opportunistic pathogen. When infecting a host, it can generally be recovered from the respiratory tract, the urogenital tract, or conjunctiva from the host: common hosts include mice, rats, hamsters, guinea pigs, rabbits, cats, and other laboratory animals. In the case of humans, many strains from *Pasteurella multocida* subsp. multocida, Pasteurella multocida subsp. septica, *Pasteurella canis*, *Pasteurella stomatis*, and Pasteurella dogmatis have been isolated from infected humans. Toxins were produced only by one strain of *P. multocida* subsp. multocida and P. canis; in addition, other than one severe case of necrotizing cellulitits caused by *P. dagmatis*, *P. multocida* subsp. multocida or *P. multocida* subsp. septica was involved in the more serious cases of infection. Symptoms of a *Pasteurella* infection vary on which body organ is involved and how long the disease is present. One of the most common symptoms is during respiratory infection and manifests as a nasal discharge. Others include sneezing, congestion, conjunctivitis, and clogged tear ducts. *Pasteurella* infections also can cause abscesses under the skin that can be chronic. Some abscesses can go as far as causing central nervous symptoms."	https://microbewiki.kenyon.edu/index.php/Pasteurella
	Actinopteri	***Bacteroides***	"*Bacteroides* are commonly found in the human intestine where they have a symbiotic host-bacterial relationship with humans. They assist in breaking down food and producing valuable nutrients and energy that the body needs. However, when Bacteriodes are introduced to parts of the body other than the gastrointestinal area, they can cause or exacerbate abscesses and other infections."<br>"Western humans share substantial portions of their microbiome that are distinct from those of non-Western humans and the nonhuman primates, including 508 conserved clades from the genus *Bacteroides*, 107 from *Ruminococcus*, and 54 from *Akkermansia*. These results agree with previous reports that indicate that diets rich in fats, such as the standard Western diet, support microbiomes high in *Bacteroides*."	Patric S.(2015) Bacteroides.Chapter 51. In: Molecular Medical Microbiology (Second Edition). Pages 917-944.<br>Gaulke CA, Arnold HK, Humphreys IR et al. Ecophylogenetics clarifies the evolutionary association between mammals and their gut microbiota. *mBio*, 2018;9:e01348–18.
	Sauropsida	*Bacteroides*	*i.b.i.d*	*i.b.i.d*
	Aves	*Lactobacillales*	"It is one of the normal flora in the oral cavity, intestine and vagina of human and animals, rarely causes disease, except for very occasionally causing subacute bacterial endocarditis, basically harmless to human. *Lactobacillus* parasiticus in the oral cavity plays an important role in the occurrence of dental caries. It is generally believed that Lactobacillus parasiticus in the intestine and vagina has a protective effect on the organism."	Tanprasertsuk, J., Shmalberg, J., Maughan, H., et al. (2021). Heterogeneity of gut microbial responses in healthy household dogs transitioning from an extruded to a mildly cooked diet. PeerJ, 9, e11648. https://doi.org/10.7717/peerj.11648
	Mammalia	*B_469493*		
Diet types	*Carnivore*	*Peptoniphilus*	"*Peptoniphilus asaccharolyticus* are gram-positive anaerobic cocci (GPAC) usually found as commensals of the skin or in the setting of polymicrobial colonization of chronic wounds."	Müller-Schulte, E., Heimann, K. C., & Treder, W. (2019). Peptoniphilus asaccharolyticus - Commensal, pathogen or synergist? Two case reports on invasive Peptoniphilus asaccharolyticus infection. Anaerobe, 59, 159–162. https://doi.org/10.1016/j.anaerobe.2019.07.001
	Herbivore	*Pasteurellales*	"*Pasteurella pneumotropica* is an opportunistic pathogen. When infecting a host, it can generally be recovered from the respiratory tract, the urogenital tract, or conjunctiva from the host: common hosts include mice, rats, hamsters, guinea pigs, rabbits, cats, and other laboratory animals. In the case of humans, many strains from *Pasteurella multocida* subsp. multocida, Pasteurella multocida subsp. septica, *Pasteurella canis*, *Pasteurella stomatis*, and Pasteurella dogmatis have been isolated from infected humans. Toxins were produced only by one strain of *P. multocida* subsp. multocida and P. canis; in addition, other than one severe case of necrotizing cellulitits caused by *P. dagmatis*, *P. multocida* subsp. multocida or *P. multocida* subsp. septica was involved in the	https://microbewiki.kenyon.edu/index.php/Pasteurella

			more serious cases of infection. Symptoms of a *Pasteurella* infection vary on which body organ is involved and how long the disease is present. One of the most common symptoms is during respiratory infection and manifests as a nasal discharge. Others include sneezing, congestion, conjunctivitis, and clogged tear ducts. *Pasteurella* infections also can cause abscesses under the skin that can be chronic. Some abscesses can go as far as causing central nervous symptoms."	
	Omnivore	*Wolbachia*	"*Wolbachia* are Gram-negative bacteria. Although a great deal is known about their symbiotic relationships, other characteristics are difficult to study, because these bacteria are difficult to cultivate independently of their host organisms."	Landmann F. (2019). The Wolbachia Endosymbionts. Microbiology Spectrum, 7(2), 10.1128/microbiolspec.BAI-0018-2019.
Vertebrates *vs.* Invertebrates	*Invertebrates*	*Pasteurellales*	"*Pasteurella pneumotropica* is an opportunistic pathogen. When infecting a host, it can generally be recovered from the respiratory tract, the urogenital tract, or conjunctiva from the host: common hosts include mice, rats, hamsters, guinea pigs, rabbits, cats, and other laboratory animals. In the case of humans, many strains from *Pasteurella multocida* subsp. multocida, Pasteurella multocida subsp. septica, *Pasteurella canis*, *Pasteurella stomatis*, and Pasteurella dogmatis have been isolated from infected humans. Toxins were produced only by one strain of *P. multocida* subsp. multocida and P. canis; in addition, other than one severe case of necrotizing cellulitits caused by *P. dagmatis*, *P. multocida* subsp. multocida or *P. multocida* subsp. septica was involved in the more serious cases of infection. Symptoms of a *Pasteurella* infection vary on which body organ is involved and how long the disease is present. One of the most common symptoms is during respiratory infection and manifests as a nasal discharge. Others include sneezing, congestion, conjunctivitis, and clogged tear ducts. *Pasteurella* infections also can cause abscesses under the skin that can be chronic. Some abscesses can go as far as causing central nervous symptoms."	https://microbewiki.kenyon.edu/index.php/Pasteurella
	Vertebrates	***Bacteroides***	"*Bacteroides* are commonly found in the human intestine where they have a symbiotic host-bacterial relationship with humans. They assist in breaking down food and producing valuable nutrients and energy that the body needs. However, when Bacteriodes are introduced to parts of the body other than the gastrointestinal area, they can cause or exacerbate abscesses and other infections."<br>"Western humans share substantial portions of their microbiome that are distinct from those of non-Western humans and the nonhuman primates, including 508 conserved clades from the genus *Bacteroides*, 107 from *Ruminococcus*, and 54 from *Akkermansia*. These results agree with previous reports that indicate that diets rich in fats, such as the standard Western diet, support microbiomes high in *Bacteroides*."	Patric S.(2015) Bacteroides.Chapter 51. In: Molecular Medical Microbiology (Second Edition). Pages 917-944.<br>Gaulke CA, Arnold HK, Humphreys IR et al. Ecophylogenetics clarifies the evolutionary association between mammals and their gut microbiota. *mBio*, 2018;9:e01348–18.
Species	*Apis mellifera*	*B_462946*		
	Bos taurus	*Treponema*	Treponema pallidum is the causative agent of syphilis.	
All combined	*All*	*Enterobacteriaceae*	"It has a wide distribution and a large host range, and is parasitic or symbiotic, epiphytic and saprophytic in humans, animals and plants, and can also survive in soil or water, and is closely related to humans."	Magruder, M., Edusei, E., Zhang, L., et al. (2020). Gut commensal microbiota and decreased risk for Enterobacteriaceae bacteriuria and urinary tract infection. Gut Microbes, 12(1), 1805281.
The 22 AGM networks for 22 Animal Orders				
Chromadorea	*Rhabditida*	*Sphingomonas*	"Some species of Sphingomonas (especially S. paucimobilis) cause minor infection in humans and damage some other organisms."	Lombardino, J., Bijlani, S., Singh, N. K., et al. (2022). Genomic Characterization of Potential Plant Growth-Promoting Features of Sphingomonas Strains Isolated from the International Space Station. Microbiology Spectrum, 10(1), e0199421.
Malacostraca	*Amphipoda*	*Sphingomonas yabuuchiae*	"*Sphingomonas yabuuchiae* is unique in its possession of ubiquinone 10 as its major respiratory quinone, and of glycosphingolipids (GDLs) instead of lipopolysaccharide in their cell envelopes. The bacterium is metabolically versatile, which means it can utilize a wide range of naturally occurring compounds as well as some types of environmental contaminants. Therefore, studies have been held to further explore its metabolic mechanisms for more application in biotechnology, in addition to its current utilization in biomeditation and in the food technology. Also some species of Sphingomonas (especially S. paucimobilis) cause minor infection in humans and damage some other organisms."	Raghunandan, K., Kumar, A., Kumar, S., et al. (2018). Production of gellan gum, an exopolysaccharide, from biodiesel-derived waste glycerol by Sphingomonas spp. 3 Biotech, 8(1), 71.
Insecta	*Blattodea*	*X.11187*		
	Diptera	*Gluconobacter*	"*Gluconobacter oxydans* strains are non-pathogenic to humans or animals, but they cause bacterial rot to apples and pears turning them shades of brown."	https://microbewiki.kenyon.edu/index.php/Gluconobacter_oxydans

	Hymenoptera	*Pasteurellales*	"*Pasteurella pneumotropica* is an opportunistic pathogen. When infecting a host, it can generally be recovered from the respiratory tract, the urogenital tract, or conjunctiva from the host: common hosts include mice, rats, hamsters, guinea pigs, rabbits, cats, and other laboratory animals. In the case of humans, many strains from *Pasteurella multocida* subsp. multocida, Pasteurella multocida subsp. septica, *Pasteurella canis*, *Pasteurella stomatis,* and Pasteurella dogmatis have been isolated from infected humans. Toxins were produced only by one strain of *P. multocida* subsp. multocida and P. canis; in addition, other than one severe case of necrotizing cellulitits caused by *P. dagmatis*, *P. multocida* subsp. multocida or *P. multocida* subsp. septica was involved in the more serious cases of infection. Symptoms of a *Pasteurella* infection vary on which body organ is involved and how long the disease is present. One of the most common symptoms is during respiratory infection and manifests as a nasal discharge. Others include sneezing, congestion, conjunctivitis, and clogged tear ducts. *Pasteurella* infections also can cause abscesses under the skin that can be chronic. Some abscesses can go as far as causing central nervous symptoms."	https://microbewiki.kenyon.edu/index.php/Pasteurella
	Lepidoptera	*Shewanella algae*	"It is known to cause peritonitis and obstructive pneumonia, rupture of aortic aneurysm, ear infections, bacteraemia, rare occurrences of skin and soft tissue infections , bone and joint infections, and many others"	Myung, D.S., et al. Primary Shewanella algae Bacteremia mimicking Vibrio Septicemia. Journal of Korean Medical Science, 2009. Volume 24(6): 1192-1194.
	Orthoptera	*Christensenellaceae*	"*Christensenella* in the human gut microbiome is associated with several health-promoting effects, and is believed to be strongly influenced by the genetic makeup of its host. The presence or absence of *Christensenella* is thought to impact an individual's risk of obesity. Associations with lean body type, lower BMI and reduced gain of fat tissue. In particular, *C. minuta* has been studied in obesity prevention and as a weight-loss aid."	Alonso, B. L., Irigoyen von Sierakowski, A., Sáez Nieto, et al. (2017). First report of human infection by Christensenella minuta, a gram-negative, strickly [sic] anaerobic rod that inhabits the human intestine. Anaerobe, 44, 124–125.
Actinopteri	*Cypriniformes*	*Bacteroides*	*"Bacteroides* are commonly found in the human intestine where they have a symbiotic host-bacterial relationship with humans. They assist in breaking down food and producing valuable nutrients and energy that the body needs. However, when Bacteriodes are introduced to parts of the body other than the gastrointestinal area, they can cause or exacerbate abscesses and other infections."<br>"Western humans share substantial portions of their microbiome that are distinct from those of non-Western humans and the nonhuman primates, including 508 conserved clades from the genus *Bacteroides*, 107 from *Ruminococcus*, and 54 from *Akkermansia.* These results agree with previous reports that indicate that diets rich in fats, such as the standard Western diet, support microbiomes high in *Bacteroides*."	Patric S.(2015) Bacteroides.Chapter 51. In: Molecular Medical Microbiology (Second Edition). Pages 917-944.<br>Gaulke CA, Arnold HK, Humphreys IR et al. Ecophylogenetics clarifies the evolutionary association between mammals and their gut microbiota. *mBio*, 2018;9:e01348–18.
	Salmoniformes	*Peptoniphilus*	"Peptoniphilus asaccharolyticus are gram-positive anaerobic cocci (GPAC) usually found as commensals of the skin or in the setting of polymicrobial colonisation of chronic wounds."	Müller-Schulte, E., Heimann, K. C., & Treder, W. (2019). Peptoniphilus asaccharolyticus - Commensal, pathogen or synergist? Two case reports on invasive Peptoniphilus asaccharolyticus infection. Anaerobe, 59, 159–162. https://doi.org/10.1016/j.anaerobe.2019.07.001
	Cichliformes	*Cetobacterium somerae*	"*Cetobacterium somerae* is a highly abundant bacterium in the intestinal flora of fish."	LaFrentz, B. R., LaFrentz, S. A., Beck, B. H., et al. (2020). Draft Genome Sequences of Cetobacterium somerae 2G Large and Two Novel Cetobacterium Isolates from Intestines of Channel Catfish (Ictalurus punctatus). Microbiology resource announcements, 9(44), e01006-20.
Sauropsida	*Squamata*	*OD1*		
Aves	*Anseriformes*	*Prevotella*	*"Prevotella sp*. are among the most numerous microbes culturable from the rumen and hind gut of cattle and sheep, where they help the breakdown of protein and carbohydrate foods. They are also present in humans, where they can be opportunistic pathogens. Prevotella, credited interchangeably with *Bacteroides melaninogenicus*, has been a problem for dentists for years. As a human pathogen known for creating periodontal and tooth problems, Prevotella has long been studied in order to counteract its pathogenesis."<br>"*Prevotella* contribute to intestinal health by serving as an energy source for host tissue, regulating inflammation, and promoting motility and blood flow."<br>They are widespread and conserved clades present in the mammalian gut.	Pavillion. 2000. Guide To Bacteria Associated with Infections In Humans. "Prevotella."<br><br>Gaulke CA, Arnold HK, Humphreys IR et al. Ecophylogenetics clarifies the evolutionary association between mammals and their gut microbiota. *Mbio* 2018;9:e01348–18.
	Columbiformes	*Corynebacterium*	*"Corynebacterium diphtheriae* is the etiological agent of diphtheria, an upper respiratory disease mainly affecting children. The virulence factors (most specifically diphtheria toxin) have been studied extensively and are well understood."	Hennart, M., Panunzi, L. G., Rodrigues, C., et al. (2020). Population genomics and antimicrobial resistance in Corynebacterium diphtheriae. Genome Medicine, 12(1), 107.
	Passeriformes	*Lactobacillales*	"It is one of the normal flora in the oral cavity, intestine and vagina of human and animals, rarely causes disease, except for very occasionally causing subacute bacterial endocarditis, basically harmless to human. Lactobacillus parasiticus in the oral cavity plays an important role in the occurrence of dental caries. It is generally believed that Lactobacillus parasiticus in the intestine and vagina has a protective effect on the organism."	Tanprasertsuk, J., Shmalberg, J., Maughan, H., et al. (2021). Heterogeneity of gut microbial responses in healthy household dogs transitioning from an extruded to a mildly cooked diet. PeerJ, 9, e11648. https://doi.org/10.7717/peerj.11648

	Psittaciformes	*Brevundimonas diminuta*	"*Brevundimonas diminuta* is commonly used as a test organism for validation of sterilizing-grade membrane filters due to the small size of the bacterium"	Chattopadhyay, S., Perkins, S. D., Shaw, M., & Nichols, T. L. (2017). Evaluation of Exposure to Brevundimonas diminuta and Pseudomonas aeruginosa during Showering. *Journal of Aerosol Science*, 114, 77–93.
	Struthioniformes	*Bacteroidales*	"It may play a role in maintaining a healthy colon, as well as potentially acting as a possible probiotic to help patients recover from illnesses such as inflammatory bowel disease and Clostridium difficile infection."<br>"They are widespread and conserved clades present in the mammalian gut.These conserved clades include members of the class *Alphaproteobacteria*, order *Bacteroidales*, the family *Ruminococcaceae*, and *Prevotella*."	García-Bayona, L., Coyne, M. J., & Comstock, L. E. (2021). Mobile Type VI secretion system loci of the gut Bacteroidales display extensive intra-ecosystem transfer, multi-species spread and geographical clustering. PLoS genetics, 17(4), e1009541.<br>Gaulke CA, Arnold HK, Humphreys IR et al. Ecophylogenetics clarifies the evolutionary association between mammals and their gut microbiota. *Mbio* 2018;9:e01348–18.
Mammalia	*Carnivora*	*Pseudomonas fragi*	"*Pseudomonas* bacteria can be found in many different environments such as soil, water, and plant and animal tissue. Many different species of this bacteria are opportunistic pathogens that affect humans, animals, and plants. *Pseudomonas aeruginosa*, called the "epitome" of opportunistic pathogens, almost never infects uncompromised tissues; however, it can infect practically any type of tissue if that tissue has some type of compromised defenses".	De Filippis, F., La Storia, A., Villani, F., et al. (2018). Strain-Level Diversity Analysis of Pseudomonas fragi after In Situ Pangenome Reconstruction Shows Distinctive Spoilage-Associated Metabolic Traits Clearly Selected by Different Storage Conditions. *Applied and Environmental Microbiology*, 85(1), e02212-18.
	Chiroptera	*X.5697*		
	Cingulata	*Enterococcus*	"*Enterococci* are regular inhabitants of the bowel. The genome of E. faecalis is more than 25% exogenously acquired DNA. Enterococci are the leading cause of hospital-acquired secondary infections."	Zaheer, R., Cook, S. R., Barbieri, R., Goji, N., Cameron, A., Petkau, A., Polo, R. O., Tymensen, L., Stamm, C., Song, J., Hannon, S., Jones, T., Church, D., Booker, C. W., Amoako, K., Van Domselaar, G., Read, R. R., & McAllister, T. A. (2020). Surveillance of Enterococcus spp. reveals distinct species and antimicrobial resistance diversity across a One-Health continuum. Scientific Reports, 10(1), 3937.
	Diprotodontia	*X.7435*		
	Primates	*Coriobacteriaceae*	"The bacteria isolated from the intestine could play a role in inflammatory bowel diseases (IBD). E. mucosicola is closely related to the more recently isolated *Enterorhabdus caecimuris*, as well as *Eggerthella* species."<br>It is found in the human intestinal tract.<br>"Western humans had the greatest number of conserved as well as exclusive and conserved clades that are distinct from those of non-Western humans and the nonhuman primates."	Harmsen, H. J., Wildeboer-Veloo, A. C., et al. (2000). Development of 16S rRNA-based probes for the Coriobacterium group and the Atopobium cluster and their application for enumeration of Coriobacteriaceae in human feces from volunteers of different age groups. Applied and environmental microbiology, 66(10), 4523–4527.<br>Gaulke CA, Arnold HK, Humphreys IR et al. Ecophylogenetics clarifies the evolutionary association between mammals and their gut microbiota. *mBio*, 2018;9:e01348–18.
	Rodentia	*Bacteria_89488*		

Table S7B. The basic biological information on the MAOs (most abundance OTUs) for the 8 selected AGM networks at microbial phylum level

Taxon or Diet Types	Sub-Taxon	MAO (Phylum)	Description	Reference
Class	*Insecta*	*Proteobacteria*	"They live in the digestive tract of animals or humans, e.g. *Wolynia* in cattle are commensal bacteria, *Helicobacter* in the duodenum and *Campylobacter* in the stomach are pathogenic bacteria in humans."<br>"*Campylobacter* is commonly associated with vertebrate hosts and some are considered significant pathogens."<br>"Fish and sharks, in contrast, consistently showed high levels of colonization with levels of *Proteobacteria*. Among invertebrates, colonization by *Proteobacteria* typically predominated."	Moon, C. D., Young, W., Maclean, P. H., et al. (2018). Metagenomic insights into the roles of Proteobacteria in the gastrointestinal microbiomes of healthy dogs and cats. MicrobiologyOpen, 7(5)<br>Gilbert, M. J., Duim, B., Zomer, A. L., et al. (2019). Living in Cold Blood: Arcobacter, Campylobacter, and Helicobacter in Reptiles. Frontiers in microbiology, 10, 1086.<br>Sherrill-Mix S, McCormick K, Lauder A et al. Allometry and ecology of the bilaterian gut microbiome. *mBio*, 2018;9:e00319–18.
	Mammalia	*Firmicutes*	"*Firmicutes* of the intestine is more abundant than Bacteroidetes, leading to more efficient absorption of calories from food and thus to obesity."	Grigor'eva I. N. (2020). Gallstone Disease, Obesity and the Firmicutes/Bacteroidetes Ratio as a Possible Biomarker of Gut Dysbiosis. *Journal of Personalized Medicine*, 11(1), 13.
Diet types	*Carnivore*	*Firmicutes*	*i.b.i.d*	*i.b.i.d*
	Herbivore	*Proteobacteria*	"They live in the digestive tract of animals or humans, e.g. *Wolynia* in cattle are commensal bacteria, *Helicobacter* in the duodenum and *Campylobacter* in the stomach are pathogenic bacteria in humans."<br>"*Campylobacter* is commonly associated with vertebrate hosts and some are considered significant pathogens."<br>"Fish and sharks, in contrast, consistently showed high levels of colonization with levels of *Proteobacteria*. Among invertebrates, colonization by *Proteobacteria* typically predominated."	Moon, C. D., Young, W., Maclean, P. H., et al. (2018). Metagenomic insights into the roles of Proteobacteria in the gastrointestinal microbiomes of healthy dogs and cats. MicrobiologyOpen, 7(5)<br>Gilbert, M. J., Duim, B., Zomer, A. L., et al. (2019). Living in Cold Blood: Arcobacter, Campylobacter, and Helicobacter in Reptiles. Frontiers in microbiology, 10, 1086.<br>Sherrill-Mix S, McCormick K, Lauder A et al. Allometry and ecology of the bilaterian gut microbiome. *mBio*, 2018;9:e00319–18.
	Omnivore	*Firmicutes*	"*Firmicutes* of the intestine is more abundant than Bacteroidetes, leading to more efficient absorption of calories from food and thus to obesity."	Grigor'eva I. N. (2020). Gallstone Disease, Obesity and the Firmicutes/Bacteroidetes Ratio as a Possible Biomarker of Gut Dysbiosis. *Journal of Personalized Medicine*, 11(1), 13.
Vertebrates *vs.* Invertebrates	*Invertebrates*	*Proteobacteria*	"They live in the digestive tract of animals or humans, e.g. *Wolynia* in cattle are commensal bacteria, *Helicobacter* in the duodenum and *Campylobacter* in the stomach are pathogenic bacteria in humans."<br>"*Campylobacter* is commonly associated with vertebrate hosts and some are considered significant pathogens."	Moon, C. D., Young, W., Maclean, P. H., et al. (2018). Metagenomic insights into the roles of Proteobacteria in the gastrointestinal microbiomes of healthy dogs and cats. MicrobiologyOpen, 7(5)<br>Gilbert, M. J., Duim, B., Zomer, A. L., et al. (2019). Living in Cold Blood: Arcobacter, Campylobacter, and Helicobacter in Reptiles. Frontiers in microbiology, 10, 1086.

			"Fish and sharks, in contrast, consistently showed high levels of colonization with levels of *Proteobacteria*. Among invertebrates, colonization by *Proteobacteria* typically predominated."	Sherrill-Mix S, McCormick K, Lauder A et al. Allometry and ecology of the bilaterian gut microbiome. *mBio*, 2018;9:e00319–18.
	Vertebrates	*Firmicutes*	"*Firmicutes* of the intestine is more abundant than Bacteroidetes, leading to more efficient absorption of calories from food and thus to obesity."	Grigor'eva I. N. (2020). Gallstone Disease, Obesity and the Firmicutes/Bacteroidetes Ratio as a Possible Biomarker of Gut Dysbiosis. Journal of personalized medicine, 11(1), 13.
Species	*Bos taurus*	*Firmicutes*	*i.b.i.d*	*i.b.i.d*

Table S7C. The basic biological information on the network hubs for the 14 selected AGM networks and the 22 animal-order AGM networks, both at microbial species levels.

**Taxon or Diet Types**	**Sub-Taxon**	**Hub**	**Description**	**Reference**
The 14 Selected AGM networks for various taxa and diet types				
Class	*Chromadorea*	*Rhizobiaceae*	"It can fix nitrogen symbiotically with legumes and has a good effect on the growth of legumes."	Yang, L. L., Jiang, Z., Li, Y., et al. (2020). Plasmids Related to the Symbiotic Nitrogen Fixation Are Not Only Cooperated Functionally but Also May Have Evolved over a Time Span in Family Rhizobiaceae. Genome biology and evolution, 12(11), 2002–2014.
	Insecta	*B_462279*		
	Actinopteri	*Vagococcus*	It separates from the water or salmon.	Wallbanks, S., A. J. Martinez-Murcia, J. L. Fryer, B. A. et al. (1990). "16S RRNA Sequence Determination for Members of the Genus Carnobacterium and Related Lactic Acid Bacteria and Description of Vagococcus Salmoninarum Sp. Nov." International Journal of Systematic Bacteriology 40.3: 224-30
	Sauropsida	*Bacteroides*	"*Bacteroides* are commonly found in the human intestine where they have a symbiotic host-bacterial relationship with humans. They assist in breaking down food and producing valuable nutrients and energy that the body needs. However, when Bacteriodes are introduced to parts of the body other than the gastrointestinal area, they can cause or exacerbate abscesses and other infections."<br>"Western humans share substantial portions of their microbiome that are distinct from those of non-Western humans and the nonhuman primates, including 508 conserved clades from the genus *Bacteroides*, 107 from *Ruminococcus*, and 54 from *Akkermansia*. These results agree with previous reports that indicate that diets rich in fats, such as the standard Western diet, support microbiomes high in *Bacteroides*."	Patric S.(2015) Bacteroides.Chapter 51. In: Molecular Medical Microbiology (Second Edition). Pages 917-944.<br>Gaulke CA, Arnold HK, Humphreys IR et al. Ecophylogenetics clarifies the evolutionary association between mammals and their gut microbiota. *mBio*, 2018;9:e01348–18.
	Aves	*Enterobacteriaceae*	"It has a wide distribution and a large host range, and is parasitic or symbiotic, epiphytic and saprophytic in humans, animals and plants, and can also survive in soil or water, and is closely related to humans."	Magruder, M., Edusei, E., Zhang, L., et al. (2020). Gut commensal microbiota and decreased risk for Enterobacteriaceae bacteriuria and urinary tract infection. Gut microbes, 12(1), 1805281.
		Clostridium	"*Clostridium neonatale sp. nov*. linked to necrotizing enterocolitis in neonates and a clarification of species assignable to the genus Clostridium (Prazmowski 1880) emend."	Bernard K, Burdz T, Wiebe D, et al. Clostridium neonatale sp. nov. linked to necrotizing enterocolitis in neonates and a clarification of species assignable to the genus Clostridium (Prazmowski 1880) emend. Lawson and Rainey 2016. Int J Syst Evol Microbiol. 2018 Aug;68(8):2416-2423. doi: 10.1099/ijsem.0.002827.
	Mammalia	*Arthrobacter*	"*Arthrobacter* are your basic soil bacteria, but have been found to perform several important functions as we continue to poison the earth with various nasty chemicals. Recently, it has been discovered that several species of Arthrobacter can reduce hexavalent chromium, which can cause severe irritaions to humans, and they are also known to degrade agricultural pesticides."	Shen, L., Liu, Y., Allen, M. A., et al. (2021). Linking genomic and physiological characteristics of psychrophilic Arthrobacter to metagenomic data to explain global environmental distribution. Microbiome, 9(1), 136.
		Streptococcus	"*Streptococcus anginosus* in head and neck squamous cell carcinoma: implication in carcinogenesis. The *Streptococcus anginosus* group (*S. intermedius*, *S. constellatus*, and *S. anginosus*) is part of the normal oral flora, prevalent in purulent infections and highly prevalent in acute and chronic endodontic-periapical infections."	Liang X, Li H, Tian G, Li S. Dynamic microbe and molecule networks in a mouse model of colitis-associated colorectal cancer. Sci Rep. 2014 May 15;4:4985. doi: 10.1038/srep04985.

		mitochondria	"The *mitochondrion* is an extremely interesting and important organelle in eukaryotic cells. It is the only organelle (other than the nucleus, of course) that has its own DNA independent of the cell's chomosomal DNA; because of this and the fact that the organelle divides independent of the cell, the mitochondrion is thought to have once been a bacterial cell that colonized a eukaryotic cell. Among other things, it performs cellular respiration, has an eletron transport system that occurs across membranes, and produces ATP."	Zielonka, J., Joseph, J., Sikora, A., et al. Mitochondria-Targeted Triphenylphosphonium-Based Compounds: Syntheses, Mechanisms of Action, and Therapeutic and Diagnostic Applications. Chemical reviews, 117(15), 10043–10120.
		Arthrobacter	"Arthrobacter are your basic soil bacteria, but have been found to perform several important functions as we continue to poison the earth with various nasty chemicals. Recently, it has been discovered that several species of Arthrobacter can reduce hexavalent chromium, which can cause severe irritaions to humans, and they are also known to degrade agricultural pesticides."	Shen, L., Liu, Y., Allen, M. A., et al. (2021). Linking genomic and physiological characteristics of psychrophilic Arthrobacter to metagenomic data to explain global environmental distribution. Microbiome, 9(1), 136.
		Bacteroides fragilis	*"Bacteroides fragilis* is part of the normal microbiota of the human colon. Disruption of the mucosal surface either by inflammation, trauma, or surgery and spread of *Bacteroides fragilis* to the bloodstream or surrounding tissues results in clinically significant infection. *Bacteroides fragilis* infection is one of the common organisms involved in intrabdominal infection. Disruption of tissue barriers and the spread of intestinal flora into adjacent tissue causes polymicrobial infection. That is why *Bacteroides fragilis* is seldom the solitary organism of infection and usually part of other anaerobic organisms and *Enterobacteriaceae*."	
Diet types	*Carnivore*	*Vagococcus*	It separates from the water or salmon.	Wallbanks, S., A. J. Martinez-Murcia, J. L. Fryer, B. A. et al. (1990). "16S RRNA Sequence Determination for Members of the Genus Carnobacterium and Related Lactic Acid Bacteria and Description of Vagococcus Salmoninarum Sp. Nov." International Journal of Systematic Bacteriology 40.3: 224-30
	Herbivore	*Bacteroides*	*"Bacteroides* are commonly found in the human intestine where they have a symbiotic host-bacterial relationship with humans. They assist in breaking down food and producing valuable nutrients and energy that the body needs. However, when Bacteriodes are introduced to parts of the body other than the gastrointestinal area, they can cause or exacerbate abscesses and other infections."<br>"Western humans share substantial portions of their microbiome that are distinct from those of non-Western humans and the nonhuman primates, including 508 conserved clades from the genus *Bacteroides*, 107 from *Ruminococcus*, and 54 from *Akkermansia*. These results agree with previous reports that indicate that diets rich in fats, such as the standard Western diet, support microbiomes high in *Bacteroides*."	Patric S.(2015) Bacteroides.Chapter 51. In: Molecular Medical Microbiology (Second Edition). Pages 917-944.<br>Gaulke CA, Arnold HK, Humphreys IR et al. Ecophylogenetics clarifies the evolutionary association between mammals and their gut microbiota. *mBio*, 2018;9:e01348–18.
	Omnivore	*Clostridiales*	"Found in other soils, sea grass roots, olive mill wastewater, snake venom, bovine and human feces. It has been shown that it is endemic, and a natural part of the gut microbiome of animals and humans. Has also been found in wounds of animals and humans, as well as abscesses and peritoneal fluid-fluid in the abdomen."<br>"It manifests patterns of codiversification with their mammalian hosts."	Djukovic, A., Garzón, M. J., Canlet, C., et al. (2022). Lactobacillus supports Clostridiales to restrict gut colonization by multidrug-resistant Enterobacteriaceae. Nature communications, 13(1), 5617.<br>Gaulke CA, Arnold HK, Humphreys IR et al. Ecophylogenetics clarifies the evolutionary association between mammals and their gut microbiota. *mBio*, 2018;9:e01348–18.
		Eubacterium biforme	*"Eubacterium biforme* was found in animal cavities, feces, animal and plant products and soil."	
		Dialister	*"D. invisus* has been associated with marginal periodontitis, caries, halitosis and apical periodontitis and is commonly isolated from endodontic infections it is consider a significant human pathogen."	Siqueira JF. 2006. Characterization of Dialister Species in Infected Root Canals. Journal of Endodontics 32:1057-1061.
		Clostridiaceae	*"Candidatus savagella*, also known as Segmented Filamentous Bacteria (SFB), is a well-studied member of the gut microbiota in mammals, fish, and birds."	Muñiz Pedrogo, D. A., Chen, J., Hillmann, B., et al. (2019). An Increased Abundance of Clostridiaceae Characterizes Arthritis in Inflammatory Bowel Disease and Rheumatoid Arthritis: A Cross-sectional Study. Inflammatory bowel diseases, 25(5), 902–913.
		Oribacterium	"These bacteria are parasitic in human nasal cavity, throat, conjunctiva, vulva and skin, etc. They are generally non-pathogenic and mostly conditionally pathogenic."	Sizova, M. V., Muller, P. A., Stancyk, et al. (2014). Oribacterium parvum sp. nov. and Oribacterium asaccharolyticum sp. nov., obligately anaerobic bacteria from the human oral cavity, and emended description of the genus Oribacterium. International

				journal of systematic and evolutionary microbiology, 64(Pt 8), 2642–2649.
		Coriobacteriaceae	"The bacteria isolated from the intestine could play a role in inflammatory bowel diseases (IBD). E. mucosicola is closely related to the more recently isolated *Enterorhabdus caecimuris*, as well as *Eggerthella* species."<br>It is found in the human intestinal tract.<br>"Western humans had the greatest number of conserved as well as exclusive and conserved clades that are distinct from those of non-Western humans and the nonhuman primates."	Harmsen, H. J., Wildeboer-Veloo, A. C., et al. (2000). Development of 16S rRNA-based probes for the Coriobacterium group and the Atopobium cluster and their application for enumeration of Coriobacteriaceae in human feces from volunteers of different age groups. Applied and environmental microbiology, 66(10), 4523–4527.<br>Gaulke CA, Arnold HK, Humphreys IR et al. Ecophylogenetics clarifies the evolutionary association between mammals and their gut microbiota. *mBio*, 2018;9:e01348–18.
Vertebrates vs. Invertebrates	*Invertebrates*	*B_462279*		
		B_462653		
	Vertebrates	*Peptoniphilus*	"*Peptoniphilus asaccharolyticus* are gram-positive anaerobic cocci (GPAC) usually found as commensals of the skin or in the setting of polymicrobial colonisation of chronic wounds."	Müller-Schulte, E., Heimann, K. C., & Treder, W. (2019). Peptoniphilus asaccharolyticus - Commensal, pathogen or synergist? Two case reports on invasive Peptoniphilus asaccharolyticus infection. Anaerobe, 59, 159–162.
Species	*Apis mellifera*	*B_462180*		
	Bos taurus	*D168*		
All combined	*All*	*Peptoniphilus*	"*Peptoniphilus asaccharolyticus* are gram-positive anaerobic cocci (GPAC) usually found as commensals of the skin or in the setting of polymicrobial colonisation of chronic wounds."	Müller-Schulte, E., Heimann, K. C., & Treder, W. (2019). Peptoniphilus asaccharolyticus - Commensal, pathogen or synergist? Two case reports on invasive Peptoniphilus asaccharolyticus infection. Anaerobe, 59, 159–162.
The 22 AGM networks for 22 Animal Orders				
Chromadorea	*Rhabditida*	*Alphaproteobacteria*	It is usually found in plants.<br>"They are widespread and conserved clades present in the mammalian gut.These conserved clades include members of the class *Alphaproteobacteria*, order *Bacteroidales*, the family *Ruminococcaceae*, and *Prevotella*."	Gaulke CA, Arnold HK, Humphreys IR et al. Ecophylogenetics clarifies the evolutionary association between mammals and their gut microbiota. *Mbio* 2018;9:e01348–18.
		Rickettsiales	"They are most abundant group of planktonic cells in marine systems and possibly the most numerous bacterium in the world."	Kowalec, M., Szewczyk, T., Welc-Falęciak, et al. (2019). Rickettsiales Occurrence and Co-occurrence in Ixodes ricinus Ticks in Natural and Urban Areas. Microbial ecology, 77(4), 890–904.
Malacostraca	*Amphipoda*	*Pirellulaceae*	"This genus of bacteria is widely distributed in nature."	
Insecta	*Blattodea*	*X.10067*		
	Diptera	*Flavobacteriaceae*	It is widely found in freshwater, seawater, soil and plants.	
	Hymenoptera	*Lactobacillus*	"*Lactobacilli* produce lactic acid and are used for many different things, including yogurt production and the maintenance of healthy intestinal microflora. *Lactobacilli* are commonly associated with the gastrointestinal tract of humans. The genome of the *Lactobacillus plantarum* has been sequenced and the genomes of several other *Lactobacilli* are underway. The goal of researchers is to better understand the roles, capabilities, and interactions of *Lactobacilli*."	Chee, W., Chew, S. Y., & Than, L. (2020). Vaginal microbiota and the potential of Lactobacillus derivatives in maintaining vaginal health. Microbial cell factories, 19(1), 203.
	Lepidoptera	*Enterobacteriaceae*	"It has a wide distribution and a large host range, and is parasitic or symbiotic, epiphytic and saprophytic in humans, animals and plants, and can also survive in soil or water, and is closely related to humans."	Magruder, M., Edusei, E., Zhang, L., et al. (2020). Gut commensal microbiota and decreased risk for Enterobacteriaceae bacteriuria and urinary tract infection. Gut microbes, 12(1), 1805281.
	Orthoptera	*Butyrivibrio*	Separated from the rumen of ruminants and occasionally from the feces of mammals; not pathogenic.	Palevich, N., Kelly, W. J., Leahy, S. C., Denman, S., Altermann, E., Rakonjac, J., & Attwood, G. T. (2019). Comparative Genomics of Rumen Butyrivibrio spp. Uncovers a Continuum of Polysaccharide-Degrading Capabilities. Applied and environmental microbiology, 86(1), e01993-19.
Actinopteri	*Cypriniformes*	*Proteocatella sphenisci*	"*Proteocatella sphenisci* has the function of degradation and metabolism."	
	Salmoniformes	*Streptococcus salivarius*	"*Streptococcus salivarius* is a probiotic in saliva."	
	Cichliformes	*Rhodobacter*	"*Rhodobacter sphaeroides* is the most-studied photosynthetic organism in terms of the structural and functional light reactions, and the metabolisms of each species generate great interest within the research community, especially in regards to renewable energy	Kyndt, J. A., Robertson, S., Shoffstall, I. B., Ramaley, R. F., & Meyer, T. E. (2022). Genome Sequence and Characterization of a Xanthorhodopsin-Containing, Aerobic Anoxygenic

			sources. *Rhodobacter* are found in freshwater or marine environments."	Phototrophic Rhodobacter Species, Isolated from Mesophilic Conditions at Yellowstone National Park. Microorganisms, 10(6), 1169.
Sauropsida	*Squamata*	*Lachnospiraceae*	"This genus is present in the intestine of most healthy individuals and may be a potentially beneficial bacterium involved in the metabolism of many carbohydrates, particularly pectin (a complex dietary fiber and prebiotic) in fruits and vegetables with a high capacity for fermentation leading to the production of acetic acid and butyric acid providing a major source of energy for the host."<br>"66 clades associated with the short-chain-fatty-acid-producing family *Lachnospiraceae* were exclusively conserved in humans and absent in the other primates."	Vacca, M., Celano, G., Calabrese, F. M., Portincasa, P., Gobbetti, M., & De Angelis, M. (2020). The Controversial Role of Human Gut Lachnospiraceae. Microorganisms, 8(4), 573.<br>Gaulke CA, Arnold HK, Humphreys IR et al. Ecophylogenetics clarifies the evolutionary association between mammals and their gut microbiota. *Mbio*, 2018;9:e01348–18.
Aves	*Anseriformes*	*Bacteroidales*	"It may play a role in maintaining a healthy colon, as well as potentially acting as a possible probiotic to help patients recover from illnesses such as inflammatory bowel disease and Clostridium difficile infection."<br>"They are widespread and conserved clades present in the mammalian gut.These conserved clades include members of the class *Alphaproteobacteria*, order *Bacteroidales*, the family *Ruminococcaceae*, and *Prevotella*."	García-Bayona, L., Coyne, M. J., & Comstock, L. E. (2021). Mobile Type VI secretion system loci of the gut Bacteroidales display extensive intra-ecosystem transfer, multi-species spread and geographical clustering. PLoS genetics, 17(4), e1009541.<br>Gaulke CA, Arnold HK, Humphreys IR et al. Ecophylogenetics clarifies the evolutionary association between mammals and their gut microbiota. *Mbio* 2018;9:e01348–18.
	Columbiformes	*Hyphomonas oceanitis*		
	Passeriformes	*Saprospiraceae*	it is useful in preventing harmful algal blooms.	
		Clostridiales	"Found in other soils, sea grass roots, olive mill wastewater, snake venom, bovine and human feces. It has been shown that it is endemic, and a natural part of the gut microbiome of animals and humans. Has also been found in wounds of animals and humans, as well as abscesses and peritoneal fluid-fluid in the abdomen."<br>"It manifests patterns of codiversification with their mammalian hosts."	Djukovic, A., Garzón, M. J., Canlet, C., et al. (2022). Lactobacillus supports Clostridiales to restrict gut colonization by multidrug-resistant Enterobacteriaceae. Nature communications, 13(1), 5617.<br>Gaulke CA, Arnold HK, Humphreys IR et al. Ecophylogenetics clarifies the evolutionary association between mammals and their gut microbiota. *mBio*, 2018;9:e01348–18.
		Ruminococcaceae	"A cellulose-degrading bacterium thought to play an important role in rumen fiber breakdown, it is capable of degrading and fermenting cellulose and hemicellulose."<br>"They are widespread and conserved clades present in the mammalian gut. These conserved clades include members of the class *Alphaproteobacteria*, order *Bacteroidales*, the family *Ruminococcaceae*, and *Prevotella*."	Gu, X., Sim, J., Lee, W. L., et al. (2021). Gut Ruminococcaceae levels at baseline correlate with risk of antibiotic-associated diarrhea. iScience, 25(1), 103644.<br>Gaulke CA, Arnold HK, Humphreys IR et al. Ecophylogenetics clarifies the evolutionary association between mammals and their gut microbiota. *Mbio* 2018;9:e01348–18.
	Psittaciformes	*Lactobacillus*	"*Lactobacilli* produce lactic acid and are used for many different things, including yogurt production and the maintenance of healthy intestinal microflora. *Lactobacilli* are commonly associated with the gastrointestinal tract of humans. The genome of the *Lactobacillus plantarum* has been sequenced and the genomes of several other *Lactobacilli* are underway. The goal of researchers is to better understand the roles, capabilities, and interactions of *Lactobacilli*."	Chee, W., Chew, S. Y., & Than, L. (2020). Vaginal microbiota and the potential of Lactobacillus derivatives in maintaining vaginal health. Microbial cell factories, 19(1), 203.
	Struthioniformes	*Lachnospiraceae*	"This genus is present in the intestine of most healthy individuals and may be a potentially beneficial bacterium involved in the metabolism of many carbohydrates, particularly pectin (a complex dietary fiber and prebiotic) in fruits and vegetables with a high capacity for fermentation leading to the production of acetic acid and butyric acid providing a major source of energy for the host."<br>"66 clades associated with the short-chain-fatty-acid-producing family Lachnospiraceae were exclusively conserved in humans and absent in the other primates."	Vacca, M., Celano, G., Calabrese, F. M., Portincasa, P., Gobbetti, M., & De Angelis, M. (2020). The Controversial Role of Human Gut Lachnospiraceae. Microorganisms, 8(4), 573.<br>Gaulke CA, Arnold HK, Humphreys IR et al. Ecophylogenetics clarifies the evolutionary association between mammals and their gut microbiota. *Mbio*, 2018;9:e01348–18.
Mammalia	*Carnivora*	*Bacteria_91313*		
	Chiroptera	*X.4556*		
	Cingulata	*Clostridium perfringens*	"Although *Clostridium perfringens* is an inhabitant of human normal intestinal flora, it is a pathogen responsible for many gastrointestinal illnesses with severity ranging from mild enterotoxaemia to fatal gas gangrene."	Mehdizadeh Gohari, I., A Navarro, M., Li, J., et al. (2021). Pathogenicity and virulence of Clostridium perfringens. Virulence, 12(1), 723–753.
	Diprotodontia	*X.6280*		
		X.7420		
	Primates	*Lactobacillaceae*	"*Lactobacilli* produce lactic acid and are used for many different things, including yogurt production and the	Chee, W., Chew, S. Y., & Than, L. (2020). Vaginal microbiota and the potential of

			maintenance of healthy intestinal microflora. *Lactobacilli* are commonly associated with the gastrointestinal tract of humans. The genome of the *Lactobacillus plantarum* has been sequenced and the genomes of several other *Lactobacilli* are underway. The goal of researchers is to better understand the roles, capabilities, and interactions of *Lactobacilli*."	Lactobacillus derivatives in maintaining vaginal health. Microbial cell factories, 19(1), 203.
	Rodentia	*Ruminococcaceae*	"A cellulose-degrading bacterium thought to play an important role in rumen fiber breakdown, it is capable of degrading and fermenting cellulose and hemicellulose." "They are widespread and conserved clades present in the mammalian gut. These conserved clades include members of the class *Alphaproteobacteria*, order *Bacteroidales*, the family *Ruminococcaceae*, and *Prevotella*."	Gu, X., Sim, J., Lee, W. L., et al. (2021). Gut Ruminococcaceae levels at baseline correlate with risk of antibiotic-associated diarrhea. iScience, 25(1), 103644. Gaulke CA, Arnold HK, Humphreys IR et al. Ecophylogenetics clarifies the evolutionary association between mammals and their gut microbiota. *Mbio* 2018;9:e01348–18.

Table 7D. The basic information on the network hubs for the 8 selected AGM networks at the microbial phylum level

Taxon or Diet Types	Sub-Taxon	Hub	Description	Reference
Class	*Insecta*	*Planctomycetes*	They are a small group of aquatic bacteria that can be found in seawater, semi-saltwater, and freshwater.	
	Mammalia	*Spirochaetes*	They were increased in abundance in the intestinal microbes of mice with inflammatory bowel disease.	Gobert, A. P., Latour, Y. L., Asim, M., et al. (2022). Protective Role of Spermidine in Colitis and Colon Carcinogenesis. Gastroenterology, 162(3), 813–827.e8. https://doi.org/10.1053/j.gastro.2021.11.005
Diet types	*Carnivore*	*Verrucomicrobia*	They are found in aquatic and soil environments, or in human feces. "*Verrucomicrobia* is a component of the human gut microbiota as it contributes to the nitrogen content of animal guts. *Verrucomicrobia* bacteria are also closely related to eukaryotes and can be essential to a healthy gut, due to its anti-inflammatory properties."	Fujio-Vejar, S., Vasquez, Y., Morales, P., et al. (2017). The Gut Microbiota of Healthy Chilean Subjects Reveals a High Abundance of the Phylum Verrucomicrobia. Frontiers in Microbiology, 8, 1221–1221.
	Herbivore	*Bacteroidetes*	"They are found in the human intestine and they have a symbiotic relationship with humans. In the intestine they can degrade cellulose. Some genera can cause meningitis in newborns."	Nkosi, B., Padayachee, T., Gront, D., et al. (2022). Contrasting Health Effects of Bacteroidetes and Firmicutes Lies in Their Genomes: Analysis of P450s, Ferredoxins, and Secondary Metabolite Clusters. International journal of molecular sciences, 23(9), 5057.
	Omnivore	*GAL15*		
		GN02		
		OP11		
		SAR406		
		SBR1093		
		WS4		
		X.Parvarchaeota.		
Vertebrates vs. Invertebrates	*Invertebrates*	*Fibrobacteres*	"They live in the rumen of ruminants and has cellulase in its periplasm to break down cellulose so that the animal can absorb it."	Ransom-Jones, E., Jones, D. L., McCarthy, A. J., et al. (2012). The Fibrobacteres: an important phylum of cellulose-degrading bacteria. Microbial ecology, 63(2), 267–281.
	Vertebrates	*Deferribacteres*		
		Verrucomicrobia	They are found in aquatic and soil environments, or in human feces. "*Verrucomicrobia* is a component of the human gut microbiota as it contributes to the nitrogen content of animal guts. *Verrucomicrobia* bacteria are also closely related to eukaryotes and can be essential to a healthy gut, due to its anti-inflammatory properties."	Fujio-Vejar, S., Vasquez, Y., Morales, P., et al. (2017). The Gut Microbiota of Healthy Chilean Subjects Reveals a High Abundance of the Phylum Verrucomicrobia. Frontiers in Microbiology, 8, 1221–1221.
Species	*Bos taurus*	*Verrucomicrobia*	*i.b.i.d*	*i.b.i.d*

Table S8A. The shared species between the top clusters of the microbial phylum-level AGM networks

Taxon or Diet Types	Shared OTU	Abundance Ratio of the former to latter taxon	Description	Reference
Insecta vs. Mammalia	*Acidobacteria*	2.735	It can multiply in the intestine, vagina, lungs, mouth, and inside, causing diarrhea and thrush.	Kalam, S., Basu, A., Ahmad, I., et al. (2020). Recent Understanding of Soil Acidobacteria and Their Ecological Significance: A Critical Review. Frontiers in microbiology, 11, 580024.
	Actinobacteria	0.499	"*Actinomyces* can mainly contribute to the decay of animal and plant remains in the soil. There are also pathogenic actinomycetes, such as *Actinomyces bovis*, which can invade tissues in the cheeks and gums when injuries occur, causing actinomycosis. The most important pathogenic actinomycetes are *Mycobacterium tuberculosis* and *Mycobacterium leprae*, which can cause tuberculosis and leprosy in humans."	Buresova-Faitova, A., Kopecky, J., Sagova-Mareckova, M., et al. (2022). Comparison of Actinobacteria communities from human-impacted and pristine karst caves. MicrobiologyOpen, 11(2), e1276.
	Bacteroidetes	0.195	"They are found in the human intestine and they have a symbiotic relationship with humans. In the intestine they can degrade cellulose. Some genera can cause meningitis in newborns."	Nkosi, B., Padayachee, T., Gront, D., et al (2022). Contrasting Health Effects of Bacteroidetes and Firmicutes Lies in Their Genomes: Analysis of P450s, Ferredoxins, and Secondary Metabolite Clusters. International journal of molecular sciences, 23(9), 5057.
	Chlamydiae	0.183	"Three types of *Chlamydia* associated with human disease are *Chlamydia psittaci*, *Chlamydia trachomatis*, and *Chlamydia pneumoniae*. All three of these *Chlamydia* can cause lung infections. *Chlamydia psittaci* can be transmitted to humans by contact and inhalation through the tissues, blood, and feces of birds infected with this *Chlamydia*, such as parrots, peacocks, chickens, ducks, and pigeons. *Chlamydia trachomatis* and *Chlamydia pneumoniae* are mainly transmitted between humans by respiratory droplets, mother-infant contact and sexual contact."	Eckert, T., Goericke-Pesch, S., Heydel, C., et al. (2019). Interaction of different Chlamydiae species with bovine spermatozoa. BMC microbiology, 19(1), 23.
	Chloroflexi	2.417	"It contains mycophylls, which can assimilate a wide range of organic acids from biotic and abiotic sources in the environment."	Schwartz, S. L., Momper, L., Rangel, L. T., et al. (2022). Novel nitrite reductase domain structure suggests a chimeric denitrification repertoire in the phylum Chloroflexi. MicrobiologyOpen, 11(1), e1258.
	Crenarchaeota	0.084	They were found in the ultramicroplankton in some oceans.	De la Vega-Ruíz, G., Domínguez-Ramírez, L., Riveros-Rosas, H., et al. (2015). New insights on the mechanism of the K(+-) independent activity of crenarchaeota pyruvate kinases. PloS One, 10(3), e0119233.
	Cyanobacteria	0.640	They can live in symbiosis with fungi, moss ferns, thalliophytes, corals and even some invertebrates. "The cyanobacteria *Merismopedia sp.* can produce lipopolysaccharides which are known to create skin irritation and gastrointestinal distress."	Cassier-Chauvat, C., Blanc-Garin, V., & Chauvat, F. (2021). Genetic, Genomics, and Responses to Stresses in Cyanobacteria: Biotechnological Implications. Genes, 12(4), 500. https://microbewiki.kenyon.edu/index.php/Merismopedia
	Deferribacteres	0.188		
	Euryarchaeota	0.179	"They contain most species of archaea, including methanogenic bacteria that can often be found in the intestines of animals, salt *Bacillus* that live at very high salt concentrations, some hyperthermophilic aerobic and anaerobic bacteria, and also marine taxa."	Deschamps, P., Zivanovic, Y., Moreira, D., et al. (2014). Pangenome evidence for extensive interdomain horizontal transfer affecting lineage core and shell genes in uncultured planktonic thaumarchaeota and euryarchaeota. Genome biology and evolution, 6(7), 1549–1563.
	FBP	5.674	"This refers to the *Firmicutes*, *Bacteroides* and *Proteobacteria*, which constitute the majority of human intestinal bacteria. After the age of seven years, the ratio between the *Bacteroides* and *Firmicute* is relatively stable, and their disruption may lead to metabolic syndromes such as obesity and diabetes."	Grigor'eva I. N. (2020). Gallstone Disease, Obesity and the Firmicutes/Bacteroidetes Ratio as a Possible Biomarker of Gut Dysbiosis. Journal of personalized medicine, 11(1), 13.
	Gemmatimonadetes	1.015	They belong to the soil microbiota.	
	Lentisphaerae	0.005	Usually, the abundance is reduced in the intestine of the elderly.	Kundu, P., Blacher, E., Elinav, E., et al. (2017). Our Gut Microbiome: The Evolving Inner Self. Cell, 171(7), 1481–1493.
	Nitrospirae	0.904	They are mostly found in aquatic plants.	
	OD1	1.106		
	Proteobacteria	3.597	"They live in the digestive tract of animals or humans, e.g. *Wolynia* in cattle are commensal bacteria, *Helicobacter* in the duodenum and *Campylobacter* in the stomach are pathogenic bacteria in humans."	Moon, C. D., Young, W., Maclean, P. H., et al. (2018). Metagenomic insights into the roles of Proteobacteria in the gastrointestinal microbiomes of healthy dogs and cats. MicrobiologyOpen, 7(5)

			"Campylobacter is commonly associated with vertebrate hosts and some are considered significant pathogens."<br>"Fish and sharks, in contrast, consistently showed high levels of colonization with levels of *Proteobacteria*. Among invertebrates, colonization by *Proteobacteria* typically predominated."	Gilbert, M. J., Duim, B., Zomer, A. L., et al. (2019). Living in Cold Blood: Arcobacter, Campylobacter, and Helicobacter in Reptiles. Frontiers in microbiology, 10, 1086.<br>Sherrill-Mix S, McCormick K, Lauder A et al. Allometry and ecology of the bilaterian gut microbiome. *mBio*, 2018;9:e00319–18.
	Spirochaetes	0.800	They were increased in abundance in the intestinal microbes of mice with inflammatory bowel disease.	Gobert, A. P., Latour, Y. L., Asim, M., et al. (2022). Protective Role of Spermidine in Colitis and Colon Carcinogenesis. Gastroenterology, 162(3), 813–827.e8.
	SR1	1.010	They can cause periodontitis.	Ul Haq, I., Brantl, S., & Müller, P. (2021). A new role for SR1 from Bacillus subtilis: regulation of sporulation by inhibition of kinA translation. Nucleic acids research, 49(18), 10589–10603.
Carnivore vs. Herbivore	*Acidobacteria*	0.011	It can multiply in the intestine, vagina, lungs, mouth, and inside, causing diarrhea and thrush.	Kalam, S., Basu, A., Ahmad, I., et al. (2020). Recent Understanding of Soil Acidobacteria and Their Ecological Significance: A Critical Review. Frontiers in microbiology, 11, 580024.
	Actinobacteria	2.669	*"Actinomyces* can mainly contribute to the decay of animal and plant remains in the soil. There are also pathogenic actinomycetes, such as *Actinomyces bovis*, which can invade tissues in the cheeks and gums when injuries occur, causing actinomycosis. The most important pathogenic actinomycetes are *Mycobacterium tuberculosis* and *Mycobacterium leprae*, which can cause tuberculosis and leprosy in humans."	Buresova-Faitova, A., Kopecky, J., Sagova-Mareckova, M., et al. (2022). Comparison of Actinobacteria communities from human-impacted and pristine karst caves. MicrobiologyOpen, 11(2), e1276.
	Armatimonadetes	0.000	"They contain certain bacteria that live in a variety of harsh and extreme environmental conditions, such as at hot springs, sulphur pools, and hot spring mouths on the sea floor."	Lee, K. C., Herbold, C. W., Dunfield, P. F., et al. (2013). Phylogenetic delineation of the novel phylum Armatimonadetes (former candidate division OP10) and definition of two novel candidate divisions. Applied and environmental microbiology, 79(7), 2484–2487.
	Bacteroidetes	0.914	"They are found in the human intestine and they have a symbiotic relationship with humans. In the intestine they can degrade cellulose. Some genera can cause meningitis in newborns."	Nkosi, B., Padayachee, T., Gront, D., et al. (2022). Contrasting Health Effects of Bacteroidetes and Firmicutes Lies in Their Genomes: Analysis of P450s, Ferredoxins, and Secondary Metabolite Clusters. International journal of molecular sciences, 23(9), 5057.
	Chlamydiae	49.072	"Three types of *Chlamydia* associated with human disease are *Chlamydia psittaci*, *Chlamydia trachomatis*, and *Chlamydia pneumoniae*. All three of these *Chlamydia* can cause lung infections. *Chlamydia psittaci* can be transmitted to humans by contact and inhalation through the tissues, blood, and feces of birds infected with this *Chlamydia*, such as parrots, peacocks, chickens, ducks, and pigeons. *Chlamydia trachomatis* and *Chlamydia pneumoniae* are mainly transmitted between humans by respiratory droplets, mother-infant contact and sexual contact."	Eckert, T., Goericke-Pesch, S., Heydel, C., et al. (2019). Interaction of different Chlamydiae species with bovine spermatozoa. BMC microbiology, 19(1), 23.
	Chlorobi	0.262	"It contains *mycophylls*, which can assimilate a wide range of organic acids from biotic and abiotic sources in the environment."	Schwartz, S. L., Momper, L., Rangel, L. T., et al. (2022). Novel nitrite reductase domain structure suggests a chimeric denitrification repertoire in the phylum Chloroflexi. MicrobiologyOpen, 11(1), e1258.
	Chloroflexi	2.762	"It contains mycophylls, which can assimilate a wide range of organic acids from biotic and abiotic sources in the environment."	Schwartz, S. L., Momper, L., Rangel, L. T., et al. (2022). Novel nitrite reductase domain structure suggests a chimeric denitrification repertoire in the phylum Chloroflexi. MicrobiologyOpen, 11(1), e1258. https://doi.org/10.1002/mbo3.1258
	Crenarchaeota	0.001	They were found in the ultramicroplankton in some oceans.	De la Vega-Ruíz, G., Domínguez-Ramírez, L., Riveros-Rosas, H., et al. (2015). New insights on the mechanism of the K(+-) independent activity of crenarchaeota pyruvate kinases. PloS one, 10(3), e0119233. https://doi.org/10.1371/journal.pone.0119233
	Cyanobacteria	4.645	They can live in symbiosis with fungi, moss ferns, thalliophytes, corals and even some invertebrates. "The cyanobacteria *Merismopedia sp.* can produce lipopolysaccharides which are known to create skin irritation and gastrointestinal distress."	Cassier-Chauvat, C., Blanc-Garin, V., & Chauvat, F. (2021). Genetic, Genomics, and Responses to Stresses in Cyanobacteria: Biotechnological Implications. Genes, 12(4), 500. https://microbewiki.kenyon.edu/index.php/Merismopedia
	Deferribacteres	4.812		
	FBP	0.034	"This refers to the *Firmicutes*, *Bacteroides* and *Proteobacteria*, which constitute the majority of human intestinal bacteria. After the age of seven years, the ratio between the *Bacteroides* and *Firmicute* is relatively stable, and their disruption	Grigor'eva I. N. (2020). Gallstone Disease, Obesity and the Firmicutes/Bacteroidetes Ratio as a Possible Biomarker of Gut Dysbiosis. Journal of personalized medicine, 11(1), 13.

			may lead to metabolic syndromes such as obesity and diabetes."	
	Fibrobacteres	0.000	"They live in the rumen of ruminants and has cellulase in its periplasm to break down cellulose so that the animal can absorb it."	Ransom-Jones, E., Jones, D. L., McCarthy, A. J., et al. (2012). The Fibrobacteres: an important phylum of cellulose-degrading bacteria. Microbial ecology, 63(2), 267–281.
	Firmicutes	1.778	"*Firmicutes* of the intestine is more abundant than *Bacteroidetes*, leading to more efficient absorption of calories from food and thus to obesity."	Grigor'eva I. N. (2020). Gallstone Disease, Obesity and the Firmicutes/Bacteroidetes Ratio as a Possible Biomarker of Gut Dysbiosis. Journal of personalized medicine, 11(1), 13.
	Gemmatimonadetes	0.213	They belong to the soil microbiota.	
	Lentisphaerae	0.231	Usually, the abundance is reduced in the intestine of the elderly.	Kundu, P., Blacher, E., Elinav, E., et al. (2017). Our Gut Microbiome: The Evolving Inner Self. Cell, 171(7), 1481–1493.
	Nitrospirae	0.069	They are mostly found in aquatic plants.	
	Planctomycetes	0.014	They are a small group of aquatic bacteria that can be found in seawater, semi-saltwater, and freshwater.	
	Proteobacteria	0.942	"They live in the digestive tract of animals or humans, e.g. *Wolynia* in cattle are commensal bacteria, *Helicobacter* in the duodenum and *Campylobacter* in the stomach are pathogenic bacteria in humans." "*Campylobacter* is commonly associated with vertebrate hosts and some are considered significant pathogens." "Fish and sharks, in contrast, consistently showed high levels of colonization with levels of *Proteobacteria*. Among invertebrates, colonization by *Proteobacteria* typically predominated."	Moon, C. D., Young, W., Maclean, P. H., et al. (2018). Metagenomic insights into the roles of Proteobacteria in the gastrointestinal microbiomes of healthy dogs and cats. MicrobiologyOpen, 7(5) Gilbert, M. J., Duim, B., Zomer, A. L., et al. (2019). Living in Cold Blood: Arcobacter, Campylobacter, and Helicobacter in Reptiles. Frontiers in microbiology, 10, 1086. Sherrill-Mix S, McCormick K, Lauder A et al. Allometry and ecology of the bilaterian gut microbiome. *mBio*, 2018;9:e00319–18.
	SR1	0.446	They can cause periodontitis.	Ul Haq, I., Brantl, S., & Müller, P. (2021). A new role for SR1 from Bacillus subtilis: regulation of sporulation by inhibition of kinA translation. Nucleic acids research, 49(18), 10589–10603.
	TM6	0.004	*TM7* are specialized epiphytes that parasitize the surface of host bacteria and are closely associated with probiotics in periodontitis and other inflammatory diseases, suggesting that they are putative pathogens.	
	Verrucomicrobia	1.122	They are found in aquatic and soil environments, or in human feces. "*Verrucomicrobia* is a component of the human gut microbiota as it contributes to the nitrogen content of animal guts. *Verrucomicrobia* bacteria are also closely related to eukaryotes and can be essential to a healthy gut, due to its anti-inflammatory properties."	Fujio-Vejar, S., Vasquez, Y., Morales, P., et al. (2017). The Gut Microbiota of Healthy Chilean Subjects Reveals a High Abundance of the Phylum Verrucomicrobia. Frontiers in Microbiology, 8, 1221–1221.
	X.Parvarchaeota.	0.500		
Invertebrates vs. Vertebrates	*Acidobacteria*	4.108	It can multiply in the intestine, vagina, lungs, mouth, and inside, causing diarrhea and thrush.	Kalam, S., Basu, A., Ahmad, I., et al. (2020). Recent Understanding of Soil Acidobacteria and Their Ecological Significance: A Critical Review. Frontiers in microbiology, 11, 580024.
	Actinobacteria	0.994	*Actinomyces* can mainly contribute to the decay of animal and plant remains in the soil. There are also pathogenic actinomycetes, such as *Actinomyces bovis*, which can invade tissues in the cheeks and gums when injuries occur, causing actinomycosis. The most important pathogenic actinomycetes are *Mycobacterium tuberculosis* and *Mycobacterium leprae*, which can cause tuberculosis and leprosy in humans.	Buresova-Faitova, A., Kopecky, J., Sagova-Mareckova, M., et al. (2022). Comparison of Actinobacteria communities from human-impacted and pristine karst caves. MicrobiologyOpen, 11(2), e1276.
	Bacteroidetes	0.565	They are found in the human intestine and they have a symbiotic relationship with humans. In the intestine they can degrade cellulose. Some genera can cause meningitis in newborns.	Nkosi, B., Padayachee, T., Gront, D., et al. (2022). Contrasting Health Effects of Bacteroidetes and Firmicutes Lies in Their Genomes: Analysis of P450s, Ferredoxins, and Secondary Metabolite Clusters. International journal of molecular sciences, 23(9), 5057.
	Chloroflexi	1.050	It contains mycophylls, which can assimilate a wide range of organic acids from biotic and abiotic sources in the environment.	Schwartz, S. L., Momper, L., Rangel, L. T., et al. (2022). Novel nitrite reductase domain structure suggests a chimeric denitrification repertoire in the phylum Chloroflexi. MicrobiologyOpen, 11(1), e1258.
	Crenarchaeota	0.325	They were found in the ultramicroplankton in some oceans.	De la Vega-Ruíz, G., Domínguez-Ramírez, L., Riveros-Rosas, H., et al. (2015). New insights on the mechanism of the K(+-) independent activity of crenarchaeota pyruvate kinases. PloS one, 10(3), e0119233.
	Cyanobacteria	0.409	They can live in symbiosis with fungi, moss ferns, thalliophytes, corals and even some invertebrates. "The cyanobacteria *Merismopedia sp.* can produce lipopolysaccharides which are known to create skin irritation and gastrointestinal distress."	Cassier-Chauvat, C., Blanc-Garin, V., & Chauvat, F. (2021). Genetic, Genomics, and Responses to Stresses in Cyanobacteria: Biotechnological Implications. Genes, 12(4), 500.

				https://microbewiki.kenyon.edu/index.php/Merismopedia
	FBP	4.888	This refers to the *Firmicutes*, *Bacteroides* and *Proteobacteria*, which constitute the majority of human intestinal bacteria. After the age of seven years, the ratio between the *Bacteroides* and *Firmicute* is relatively stable, and their disruption may lead to metabolic syndromes such as obesity and diabetes.	Grigor'eva I. N. (2020). Gallstone Disease, Obesity and the Firmicutes/Bacteroidetes Ratio as a Possible Biomarker of Gut Dysbiosis. Journal of personalized medicine, 11(1), 13.
	Firmicutes	0.281	*Firmicutes* of the intestine is more abundant than *Bacteroidetes*, leading to more efficient absorption of calories from food and thus to obesity.	Grigor'eva I. N. (2020). Gallstone Disease, Obesity and the Firmicutes/Bacteroidetes Ratio as a Possible Biomarker of Gut Dysbiosis. Journal of personalized medicine, 11(1), 13.
	Gemmatimonadetes	1.102	They belong to the soil microbiota.	
	Nitrospirae	0.566	They are mostly found in aquatic plants.	
	Planctomycetes	0.893	They are a small group of aquatic bacteria that can be found in seawater, semi-saltwater, and freshwater.	
	Proteobacteria	4.848	"They live in the digestive tract of animals or humans, e.g. *Wolynia* in cattle are commensal bacteria, *Helicobacter* in the duodenum and *Campylobacter* in the stomach are pathogenic bacteria in humans." "*Campylobacter* is commonly associated with vertebrate hosts and some are considered significant pathogens." "Fish and sharks, in contrast, consistently showed high levels of colonization with levels of *Proteobacteria*. Among invertebrates, colonization by *Proteobacteria* typically predominated."	Moon, C. D., Young, W., Maclean, P. H., et al. (2018). Metagenomic insights into the roles of Proteobacteria in the gastrointestinal microbiomes of healthy dogs and cats. MicrobiologyOpen, 7(5) Gilbert, M. J., Duim, B., Zomer, A. L., et al. (2019). Living in Cold Blood: Arcobacter, Campylobacter, and Helicobacter in Reptiles. Frontiers in microbiology, 10, 1086. Sherrill-Mix S, McCormick K, Lauder A et al. Allometry and ecology of the bilaterian gut microbiome. *mBio*, 2018;9:e00319–18.
	SBR1093	2.761		
	Spirochaetes	1.144	They were increased in abundance in the intestinal microbes of mice with inflammatory bowel disease	Gobert, A. P., Latour, Y. L., Asim, M., et al. (2022). Protective Role of Spermidine in Colitis and Colon Carcinogenesis. Gastroenterology, 162(3), 813–827.e8.
	SR1	1.079		
	TM6	0.306	*TM7* are specialized epiphytes that parasitize the surface of host bacteria and are closely associated with probiotics in periodontitis and other inflammatory diseases, suggesting that they are putative pathogens.	
	Verrucomicrobia	0.789	They are found in aquatic and soil environments, or in human feces. "*Verrucomicrobia* is a component of the human gut microbiota as it contributes to the nitrogen content of animal guts. *Verrucomicrobia* bacteria are also closely related to eukaryotes and can be essential to a healthy gut, due to its anti-inflammatory properties."	Fujio-Vejar, S., Vasquez, Y., Morales, P., et al. (2017). The Gut Microbiota of Healthy Chilean Subjects Reveals a High Abundance of the Phylum Verrucomicrobia. Frontiers in Microbiology, 8, 1221–1221.

Table S8B. The unique species in the top clusters of the microbial phylum-level AGM networks

Group	OTU	Description	Reference
Insecta	*Synergistetes*	It can cause multiple infectious diseases of the oral cavity such as periodontal disease, caries and pulpitis, and is a major factor in the destruction of periodontal tissues.	Vartoukian, S. R., Palmer, R. M., & Wade, W. G. (2009). Diversity and morphology of members of the phylum "synergistetes" in periodontal health and disease. Applied and environmental microbiology, 75(11), 3777–3786.
	Elusimicrobia	Soil bacterial community	
	TM6	*TM7* are specialized epiphytes that parasitize the surface of host bacteria and are closely associated with probiotics in periodontitis and other inflammatory diseases, suggesting that they are putative pathogens.	
	Planctomycetes	They are a small group of aquatic bacteria that can be found in seawater, semi-saltwater, and freshwater.	
Mammalia	*Firmicutes*	*Firmicutes* of the intestine is more abundant than *Bacteroidetes*, leading to more efficient absorption of calories from food and thus to obesity.	Grigor'eva I. N. (2020). Gallstone Disease, Obesity and the Firmicutes/Bacteroidetes Ratio as a Possible Biomarker of Gut Dysbiosis. Journal of personalized medicine, 11(1), 13.
	Tenericutes	"Birds showed high levels of colonization with *Firmicutes* and *Tenericutes*."	Sherrill-Mix S, McCormick K, Lauder A et al. Allometry and ecology of the bilaterian gut microbiome. *mBio* 2018;9:e00319–18.
	Verrucomicrobia	They are found in aquatic and soil environments, or in human feces. "*Verrucomicrobia* is a component of the human gut microbiota as it contributes to the nitrogen content	Fujio-Vejar, S., Vasquez, Y., Morales, P., et al. (2017). The Gut Microbiota of Healthy Chilean Subjects Reveals a High Abundance of the Phylum Verrucomicrobia. Frontiers in Microbiology, 8, 1221–1221.

		of animal guts. *Verrucomicrobia* bacteria are also closely related to eukaryotes and can be essential to a healthy gut, due to its anti-inflammatory properties."	
	Fibrobacteres	They live in the rumen of ruminants and has cellulase in its periplasm to break down cellulose so that the animal can absorb it.	Ransom-Jones, E., Jones, D. L., McCarthy, A. J., et al. (2012). The Fibrobacteres: an important phylum of cellulose-degrading bacteria. Microbial ecology, 63(2), 267–281.
	Chlorobi	It contains mycophylls, which can assimilate a wide range of organic acids from biotic and abiotic sources in the environment.	Schwartz, S. L., Momper, L., Rangel, L. T., et al. (2022). Novel nitrite reductase domain structure suggests a chimeric denitrification repertoire in the phylum Chloroflexi. MicrobiologyOpen, 11(1), e1258.
	Armatimonadetes	They contain certain bacteria that live in a variety of harsh and extreme environmental conditions, such as at hot springs, sulphur pools, and hot spring mouths on the sea floor.	
	X.Thermi.	It exists in acidic high temperature environments.	Benammar, L., İnan Bektaş, K., Menasria, T., et al. (2020). Diversity and enzymatic potential of thermophilic bacteria associated with terrestrial hot springs in Algeria. Brazilian journal of microbiology : [publication of the Brazilian Society for Microbiology], 51(4), 1987–2007.
	X.Parvarchaeota.		
	Thermotogae	It is a group of thermophilic or hyperthermophilic bacteria.	
	Synergistetes	It can cause multiple infectious diseases of the oral cavity such as periodontal disease, caries and pulpitis, and is a major factor in the destruction of periodontal tissues.	Vartoukian, S. R., Palmer, R. M., & Wade, W. G. (2009). Diversity and morphology of members of the phylum "synergistetes" in periodontal health and disease. Applied and environmental microbiology, 75(11), 3777–3786.
	Fusobacteria	It is normally parasitic in the oral cavity, upper gastrointestinal tract, intestinal and genitourinary tracts and soil of humans or animals, and is most common in oral tartar.	Park, C. H., Han, D. S., Oh, Y. et al. (2016). Role of Fusobacteria in the serrated pathway of colorectal carcinogenesis. Scientific reports, 6, 25271.
	Spirochaetes	They were increased in abundance in the intestinal microbes of mice with inflammatory bowel disease	Gobert, A. P., Latour, Y. L., Asim, M., et al. (2022). Protective Role of Spermidine in Colitis and Colon Carcinogenesis. Gastroenterology, 162(3), 813–827.e8.
	Euryarchaeota	They contain most species of archaea, including methanogenic bacteria that can often be found in the intestines of animals, salt *Bacillus* that live at very high salt concentrations, some hyperthermophilic aerobic and anaerobic bacteria, and also marine taxa.	Deschamps, P., Zivanovic, Y., Moreira, D., et al. (2014). Pangenome evidence for extensive interdomain horizontal transfer affecting lineage core and shell genes in uncultured planktonic thaumarchaeota and euryarchaeota. Genome biology and evolution, 6(7), 1549–1563.
	Tenericutes	"Birds showed high levels of colonization with *Firmicutes* and *Tenericutes*."	Sherrill-Mix S, McCormick K, Lauder A et al. Allometry and ecology of the bilaterian gut microbiome. *mBio* 2018;9:e00319–18.
	X.Thermi.	It exists in acidic high temperature environments.	Benammar, L., İnan Bektaş, K., Menasria, T., et al. (2020). Diversity and enzymatic potential of thermophilic bacteria associated with terrestrial hot springs in Algeria. Brazilian journal of microbiology : [publication of the Brazilian Society for Microbiology], 51(4), 1987–2007.
Invertebrates	*Fusobacteria*	It is normally parasitic in the oral cavity, upper gastrointestinal tract, intestinal and genitourinary tracts and soil of humans or animals, and is most common in oral tartar.	Park, C. H., Han, D. S., Oh, Y. et al. (2016). Role of Fusobacteria in the serrated pathway of colorectal carcinogenesis. Scientific reports, 6, 25271.
	Fibrobacteres	They live in the rumen of ruminants and has cellulase in its periplasm to break down cellulose so that the animal can absorb it.	Ransom-Jones, E., Jones, D. L., McCarthy, A. J., et al. (2012). The Fibrobacteres: an important phylum of cellulose-degrading bacteria. Microbial ecology, 63(2), 267–281.
	Elusimicrobia	Soil bacterial community	
	Lentisphaerae	Usually, the abundance is reduced in the intestine of the elderly.	Kundu, P., Blacher, E., Elinav, E., et al. (2017). Our Gut Microbiome: The Evolving Inner Self. Cell, 171(7), 1481–1493.
	Synergistetes	It can cause multiple infectious diseases of the oral cavity such as periodontal disease, caries and pulpitis, and is a major factor in the destruction of periodontal tissues.	Vartoukian, S. R., Palmer, R. M., & Wade, W. G. (2009). Diversity and morphology of members of the phylum "synergistetes" in periodontal health and disease. Applied and environmental microbiology, 75(11), 3777–3786.
Vertebrates	*Chlorobi*	It contains mycophylls, which can assimilate a wide range of organic acids from biotic and abiotic sources in the environment.	Schwartz, S. L., Momper, L., Rangel, L. T., et al. (2022). Novel nitrite reductase domain structure suggests a chimeric denitrification repertoire in the phylum Chloroflexi. MicrobiologyOpen, 11(1), e1258.
	Tenericutes	"Birds showed high levels of colonization with *Firmicutes* and *Tenericutes*."	Sherrill-Mix S, McCormick K, Lauder A et al. Allometry and ecology of the bilaterian gut microbiome. *mBio* 2018;9:e00319–18.
	Chlamydiae	Three types of *Chlamydia* associated with human disease are *Chlamydia psittaci*, *Chlamydia trachomatis*, and *Chlamydia pneumoniae*. All three of these *Chlamydia* can cause lung infections. *Chlamydia psittaci* can be transmitted to humans by contact and inhalation through the tissues, blood, and feces of birds infected with this *Chlamydia*, such as parrots, peacocks, chickens, ducks, and pigeons. *Chlamydia trachomatis* and *Chlamydia pneumoniae* are mainly transmitted between humans by respiratory droplets, mother-infant contact and sexual contact.	Eckert, T., Goericke-Pesch, S., Heydel, C., et al. (2019). Interaction of different Chlamydiae species with bovine spermatozoa. BMC microbiology, 19(1), 23.
	Deferribacteres		

	Armatimonadetes	They contain certain bacteria that live in a variety of harsh and extreme environmental conditions, such as at hot springs, sulphur pools, and hot spring mouths on the sea floor.	
	WWE1		
	Thermotogae	It is a group of thermophilic or hyperthermophilic bacteria.	
	Aquificae	A class of bacteria that live in environments such as hot springs, sulphur pools, and hot spring mouths on the seabed.	
	Euryarchaeota	They contain most species of archaea, including methanogenic bacteria that can often be found in the intestines of animals, salt *Bacillus* that live at very high salt concentrations, some hyperthermophilic aerobic and anaerobic bacteria, and also marine taxa.	Deschamps, P., Zivanovic, Y., Moreira, D., et al. (2014). Pangenome evidence for extensive interdomain horizontal transfer affecting lineage core and shell genes in uncultured planktonic thaumarchaeota and euryarchaeota. Genome biology and evolution, 6(7), 1549–1563.
	X.Thermi.	It exists in acidic high temperature environments.	Benammar, L., İnan Bektaş, K., Menasria, T., et al. (2020). Diversity and enzymatic potential of thermophilic bacteria associated with terrestrial hot springs in Algeria. Brazilian journal of microbiology : [publication of the Brazilian Society for Microbiology], 51(4), 1987–2007.

Table S8C. The biological information on the nodes of the microbial phylum-level AGM networks for the order of *primate*.

OTU	Description	Reference
Actinobacteria	*"Actinomyces* can mainly contribute to the decay of animal and plant remains in the soil. There are also pathogenic actinomycetes, such as *Actinomyces bovis*, which can invade tissues in the cheeks and gums when injuries occur, causing actinomycosis. The most important pathogenic actinomycetes are *Mycobacterium tuberculosis* and *Mycobacterium leprae*, which can cause tuberculosis and leprosy in humans."	Buresova-Faitova, A., Kopecky, J., Sagova-Mareckova, M., et al. (2022). Comparison of Actinobacteria communities from human-impacted and pristine karst caves. MicrobiologyOpen, 11(2), e1276.
Bacteroidetes	"They are found in the human intestine and they have a symbiotic relationship with humans. In the intestine they can degrade cellulose. Some genera can cause meningitis in newborns."	Nkosi, B., Padayachee, T., Gront, D., et al. (2022). Contrasting Health Effects of Bacteroidetes and Firmicutes Lies in Their Genomes: Analysis of P450s, Ferredoxins, and Secondary Metabolite Clusters. International journal of molecular sciences, 23(9), 5057.
Cyanobacteria	They can live in symbiosis with fungi, moss ferns, thalliophytes, corals and even some invertebrates.<br>"The cyanobacteria *Merismopedia sp.* can produce lipopolysaccharides which are known to create skin irritation and gastrointestinal distress."	Cassier-Chauvat, C., Blanc-Garin, V., & Chauvat, F. (2021). Genetic, Genomics, and Responses to Stresses in Cyanobacteria: Biotechnological Implications. Genes, 12(4), 500.<br>https://microbewiki.kenyon.edu/index.php/Merismopedia
Firmicutes	*"Firmicutes* of the intestine is more abundant than *Bacteroidetes*, leading to more efficient absorption of calories from food and thus to obesity."	Grigor'eva I. N. (2020). Gallstone Disease, Obesity and the Firmicutes/Bacteroidetes Ratio as a Possible Biomarker of Gut Dysbiosis. Journal of personalized medicine, 11(1), 13.
Proteobacteria	"They live in the digestive tract of animals or humans, e.g. *Wolynia* in cattle are commensal bacteria, *Helicobacter* in the duodenum and *Campylobacter* in the stomach are pathogenic bacteria in humans."<br>*"Campylobacter* is commonly associated with vertebrate hosts and some are considered significant pathogens."<br>"Fish and sharks, in contrast, consistently showed high levels of colonization with levels of *Proteobacteria*. Among invertebrates, colonization by *Proteobacteria* typically predominated."	Moon, C. D., Young, W., Maclean, P. H., et al. (2018). Metagenomic insights into the roles of Proteobacteria in the gastrointestinal microbiomes of healthy dogs and cats. MicrobiologyOpen, 7(5)<br>Gilbert, M. J., Duim, B., Zomer, A. L., et al. (2019). Living in Cold Blood: Arcobacter, Campylobacter, and Helicobacter in Reptiles. Frontiers in microbiology, 10, 1086.<br>Sherrill-Mix S, McCormick K, Lauder A et al. Allometry and ecology of the bilaterian gut microbiome. *mBio*, 2018;9:e00319–18.
Spirochaetes	They were increased in abundance in the intestinal microbes of mice with inflammatory bowel disease	Gobert, A. P., Latour, Y. L., Asim, M., et al. (2022). Protective Role of Spermidine in Colitis and Colon Carcinogenesis. Gastroenterology, 162(3), 813–827.e8.
Tenericutes	"Birds showed high levels of colonization with *Firmicutes* and *Tenericutes*."	Sherrill-Mix S, McCormick K, Lauder A et al. Allometry and ecology of the bilaterian gut microbiome. *mBio* 2018;9:e00319–18.
Verrucomicrobia	They are found in aquatic and soil environments, or in human feces. "*Verrucomicrobia* is a component of the human gut microbiota as it contributes to the nitrogen content of animal guts. *Verrucomicrobia* bacteria are also closely related to eukaryotes and can be essential to a healthy gut, due to its anti-inflammatory properties."	Fujio-Vejar, S., Vasquez, Y., Morales, P., et al. (2017). The Gut Microbiota of Healthy Chilean Subjects Reveals a High Abundance of the Phylum Verrucomicrobia. Frontiers in Microbiology, 8, 1221–1221.
